# Short Time-Scale Sensory Coding in S1 during Discrimination of Whisker Vibrotactile Sequences

**DOI:** 10.1371/journal.pbio.1002549

**Published:** 2016-08-30

**Authors:** Leah M. McGuire, Gregory Telian, Keven J. Laboy-Juárez, Toshio Miyashita, Daniel J. Lee, Katherine A. Smith, Daniel E. Feldman

**Affiliations:** Department of Molecular and Cellular Biology, and Helen Wills Neuroscience Institute, University of California Berkeley, Berkeley, California, United States of America; Ecole Polytechnique Federale de Lausanne, SWITZERLAND

## Abstract

Rodent whisker input consists of dense microvibration sequences that are often temporally integrated for perceptual discrimination. Whether primary somatosensory cortex (S1) participates in temporal integration is unknown. We trained rats to discriminate whisker impulse sequences that varied in single-impulse kinematics (5–20-ms time scale) and mean speed (150-ms time scale). Rats appeared to use the integrated feature, mean speed, to guide discrimination in this task, consistent with similar prior studies. Despite this, 52% of S1 units, including 73% of units in L4 and L2/3, encoded sequences at fast time scales (≤20 ms, mostly 5–10 ms), accurately reflecting single impulse kinematics. 17% of units, mostly in L5, showed weaker impulse responses and a slow firing rate increase during sequences. However, these units did not effectively integrate whisker impulses, but instead combined weak impulse responses with a distinct, slow signal correlated to behavioral choice. A neural decoder could identify sequences from fast unit spike trains and behavioral choice from slow units. Thus, S1 encoded fast time scale whisker input without substantial temporal integration across whisker impulses.

## Abstract 2

Recordings in whisker somatosensory cortex of rats during discrimination of rapid whisker deflection sequences show that whisker input is encoded at very short time scales (less than 20 ms).

## Author Summary

Sensory input is rich in temporal patterns, but how the brain processes this temporal information is not well understood. This process is important in the whisker tactile system of rodents, in which active whisking on objects generates dense streams of stick-slip and contact events. Rats can discriminate vibrotactile sequences applied to the whiskers, and prior studies show that this often involves behavioral integration over time to calculate mean whisker-speed. How the brain represents and integrates vibrotactile input is not known. We recorded neural activity in primary somatosensory cortex as rats discriminated rapid vibrotactile sequences. We found that neurons in the primary somatosensory cortex encoded whisker sensory information at very fast time scales (<20 ms), without evidence for substantial temporal integration. A subset of neurons encoded relatively little stimulus information but strongly encoded the rat’s behavioral choice on each trial. Thus, primary sensory cortex represents immediate sensory input, suggesting that temporal integration occurs in downstream brain areas.

## Introduction

Natural sensory input comprises dense temporal series of discrete events, which animals often temporally integrate to guide perceptual decisions. The temporal integration process has been studied in primate somatosensation and vision [1,2], but less in rodents, in which modern tools could reveal the underlying circuit mechanisms. In the whisker tactile system, active whisking generates dense streams of stick-slip events on surfaces (5–10 ms duration, ~60 ms interval) [3,4] and contact events on object edges [5,6]. These temporal series constitute the whisker vibrotactile signal. While animals can perceive individual brief whisker impulses alone or within trains [7–11], behavioral discrimination of vibrotactile sequences is often based on a time-averaged composite feature, mean whisker speed, rather than the kinematics or precise pattern of individual deflections [12,13]. This suggests that the brain generates both short time-scale (individual impulse) and temporally integrated, long time-scale (mean speed or intensity) representations of whisker input. How these time scales are represented in the cortex is unknown.

We tested which time scale(s) of information are represented in S1 in awake behaving rats discriminating rapid whisker sequences. Under anesthesia, most S1 neurons spike phasically to whisker deflections, and responses adapt strongly during stimulus trains. This suggests that S1 does not temporally integrate across impulses (we use “integration” to mean temporal summation or averaging) [14–18]. Most S1 neurons also spike phasically to whisker deflection in basic detection tasks [7,9,10,19] or when rats must detect kinematically distinct impulses within ongoing stimulus trains [8]. However, these tasks do not require stimulus integration for behavioral performance [7–10]. Whether temporal integration occurs in S1 during tasks in which animals behaviorally integrate whisker information is unknown. A subset of S1 neurons exhibit sustained responses to stimulus sequences in awake mice [20], but whether these contribute to perceptual integration is unclear.

We trained rats to discriminate rapid sequences of three brief whisker impulses with an ~60 ms interpulse interval. This interval matches the median interval between stick-slip events during texture palpation [21]. S1 is required for passive vibrotactile discrimination [13,22,23]. Stimuli differed in both rapid temporal structure (kinematics and order of individual impulses) and time-integrated information (mean speed of the entire sequence). Rats could use either for discrimination. Behavioral choice correlated with mean speed, suggesting that rats temporally integrated whisker impulse sequences, as shown explicitly in similar prior studies in which both rapid kinematic and slow intensity cues were available [12,13]. In tetrode recordings during behavior, most S1 units accurately encoded single-impulse kinematics on a rapid (≤20 ms) time scale with modest adaptation. A minority of units responded weakly to individual impulses but exhibited slowly increasing or decreasing spiking during the stimulus period. However, these units did not effectively integrate across impulses and instead combined transient impulse responses with a distinct, slow signal correlated to behavioral choice. Thus, S1 appears to represent only short time-scale information about whisker impulse trains during vibrotactile discrimination. This suggests that temporal integration may occur downstream of S1.

## Results

### Behavioral Discrimination of FFF, FMS, SMF, and SSS Sequences

**Fig 1:**
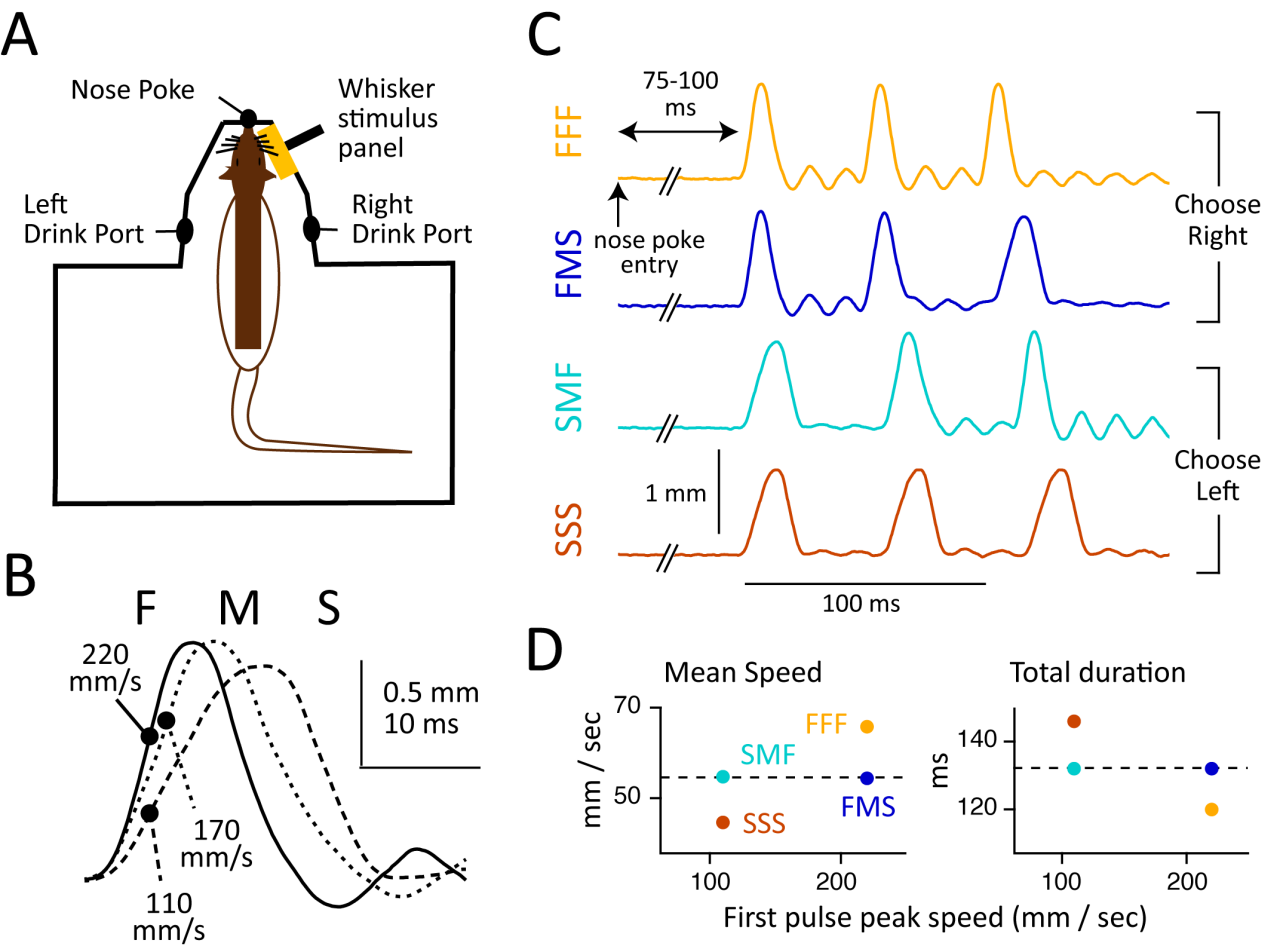
Whisker stimuli and behavioral apparatus. (A) Schematic of training apparatus, showing the rat’s right whiskers resting on the moveable stimulus panel. (B) Panel kinematics for fast, medium, and slow impulses. Circles indicate maximum velocity. (C) Panel kinematics for FFF, FMS, SMF, and SSS sequences. Data for this panel are in S1 Data. (D) Mean speed, total duration, and first pulse peak velocity for the four sequences. SMF and FMS sequences had similar mean speed and duration (dashed lines).

We developed a novel whisker vibrotactile discrimination task in which rats initiated trials by entering a nose poke with their right whiskers resting on a wall panel coupled to a hidden piezoelectric actuator (Fig 1). The panel delivered a rapid sequence of three up-down impulses. Each impulse was 16–26 ms long and had Fast (F), Medium (M), or Slow (S) rise/fall velocity. Sequences had FFF, FMS, SMF, or SSS pulse order (34 ms interval from end of a pulse to beginning of next pulse; 120–148 ms sequence duration). Sequences were constructed so that mean speed was greatest for FFF, lowest for SSS, and equal and intermediate for FMS and SMF sequences (Fig 1; Table 1; S1 Fig). One sequence was delivered per trial, beginning 75–100 ms after nose poke entry. Rats had to maintain nose poke for 250 ms to ensure delivery of the entire sequence and then discriminate by selecting a right or left drink port for water reward. FFF and FMS sequences were rewarded right, and SMF and SSS were rewarded left. Training was conducted under infrared light, and sound cues from the piezo were masked. In a subset of trials (43 trials, 4 rats), we verified with high-speed video that whiskers remained on the panel throughout the stimulus period and that rats did not whisk while in the nose poke, as shown previously [22]. Head movement averaged 0.8 mm in right-left position and 1.0 mm in rostrocaudal position during the stimulus period. Rats initially trained on FFF versus SSS discrimination and then FMS and SMF stimuli were added (see Materials and Methods).

**Table 1:** Kinematics of FFF-FMS-SMF-SSS whisker sequences.

Impulse	Rise/Fall Time (ms)	Peak Velocity (mm/s)	Duration* (ms)	Peak Amplitude (mm)
Fast (F)	8	220	16	1.03
Medium (M)	11	170	22	1.15
Slow (S)	14	110	28	1.14
Sequence	Interpulse Interval (ms)	Peak Velocity (mm/s)	Duration* (ms)	Peak amplitude (mm)
FFF	34	120	65.7	Right
FMS	34	132	54.4	Right
SMF	34	132	54.7	Left
SSS	34	148	44.6	Left

* Duration measured as time from initial deflection to return to baseline position.

These sequences differed in both rapid stimulus features, like identity of individual impulses, and slow features, like mean speed of the entire sequence. We designed the task so that fully correct discrimination is only possible if rats attend to fine time-scale information, like precise internal structure of the train (FFF or FMS indicates choose right, SMF or SSS indicates choose left), or identity of the first impulse (F indicates choose right, S indicates choose left). In contrast, if behavior is guided by mean speed (or duration) of the entire sequence, then rats should respond to FFF and SSS correctly but make mistakes in which they treat SMF and FMS identically and intermediate to FFF or SSS. Using a similar task design in which both rapid and slow, integrated cues were available, two prior studies found that rats choose to guide vibrotactile discrimination by the integrated variable, mean speed or intensity [12,13].

**Fig 2:**
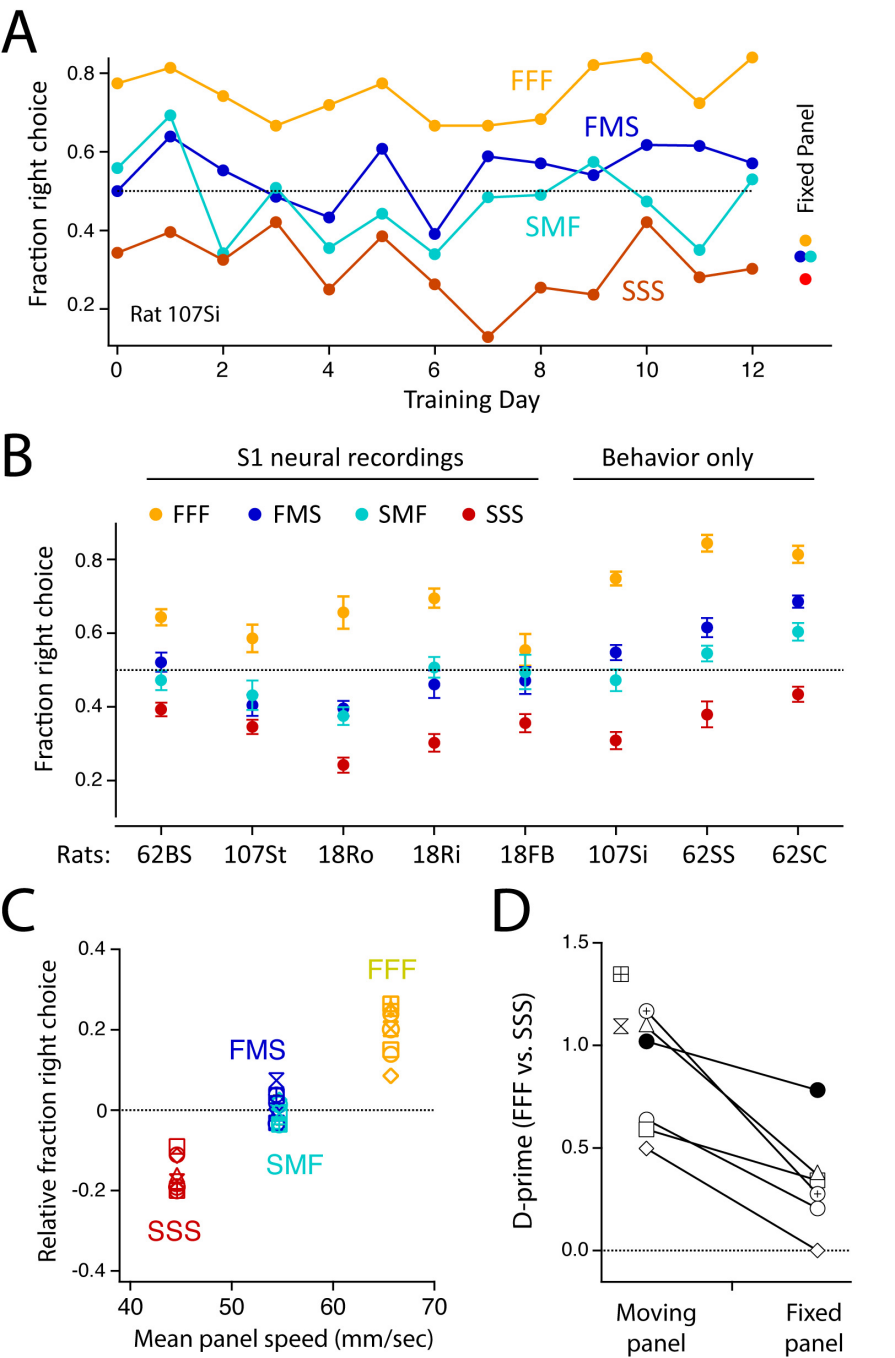
Behavioral performance on FFF-FMS-SMF-SSS discrimination task. (A) Discrimination performance for one example rat, across 13 d of training (44–50 trials for each stimulus per day). FMS and SMF stimuli were first introduced on Day 0. The rat reliably discriminated FFF from SSS stimuli but treated FMS and SMF stimuli identically and at chance. The rat responded similarly to all stimuli when the panel was fixed, and thus was not discriminating based on piezo auditory cues. (B) Mean performance (± SEM) for all rats across all behavior sessions. (C) Relative right drink port choice as a function of mean panel speed over the entire 150-ms sequence. Each symbol is a different rat (*n* = 8). (D) D-prime analysis of FFF versus SSS discrimination in fixed-panel control experiments versus normal sessions. Solitary points show rats not tested on the fixed-panel control. Data for this figure are in S1 Data.

After 14.2 ± 4.4 (standard deviation [s.d.]) (range: 8–22) d of training on FFF-FMS-SMF-SSS discrimination, all eight rats successfully discriminated FFF from SSS stimuli, but failed to respond appropriately to FMS and SMF stimuli, instead treating them as equivalent and intermediate between FFF and SSS (Fig 2A and 2B). Seven out of eight rats failed to differentiate at all between FMS from SMF stimuli (proportion test, Bonferroni-adjusted *p*-value >0.00625). One rat (62SC) showed modest but significant discrimination, with more right-side choices to FMS than SMF stimuli (*p* = 0.0039). Behavior was stable, on average, across the training period (S2 Fig). Thus, seven out of eight rats showed behavior consistent with guiding decisions by time-integrated whisker information. To examine this further, we plotted the mean behavioral performance of each rat versus the mean speed of panel movement across the entire sequence (150 ms). Behavioral performance was computed as (fraction of right drink port choices for each stimulus)–(mean fraction of right drink port choices for all stimuli), to account for right-left choice bias by some rats (Fig 2B). Right drink port choice was strongly related to mean sequence speed for all rats (Fig 2C).

To confirm that rats guided behavior by panel movement, we ran a “fixed panel” control in six rats, immediately after the final normal training session. The panel was fixed in place, while the piezo behind it moved normally. Panel fixation strongly impaired behavioral discrimination in all but one rat (example rat, Fig 2A; population data using d-prime analysis, Fig 2D; population data using a simpler non-parametric analysis, S2B Fig). Some residual discrimination did persist and may have been mediated by inadequately masked piezo sound cues. Further analysis showed that three rats treated the average fixed-panel stimulus similarly to SSS stimuli; one rat responded by choosing right or left randomly; and one rat stopped completing trials in the fixed-panel condition (S2C Fig). Thus, different rats had different strategies for handling the unfamiliar fixed panel trials.

These results suggests that, as in prior studies [12,13], rats used slow, integrated information (mean speed or intensity) to guide discrimination, rather than rapid information (first or last impulse identity or impulse order). This may reflect either a predisposition for intensity cues, or task factors such as our use of strong intensity cues in initial training or the nose poke time requirement, which may have promoted an integration-based strategy. Rats are known to sense fast kinematic cues during ongoing sequences [7–11], and they can utilize these cues for discrimination in some cases [8]. We did not apply additional stimuli to further dissociate slow from rapid information (as was done in [12,13]), and thus we cannot independently rule out the possibility that rats guided behavior from a hidden fast cue (e.g., second impulse identity) that correlated with mean speed.

### Discrimination of FSFS versus SFFS Sequences

**Fig 3:**
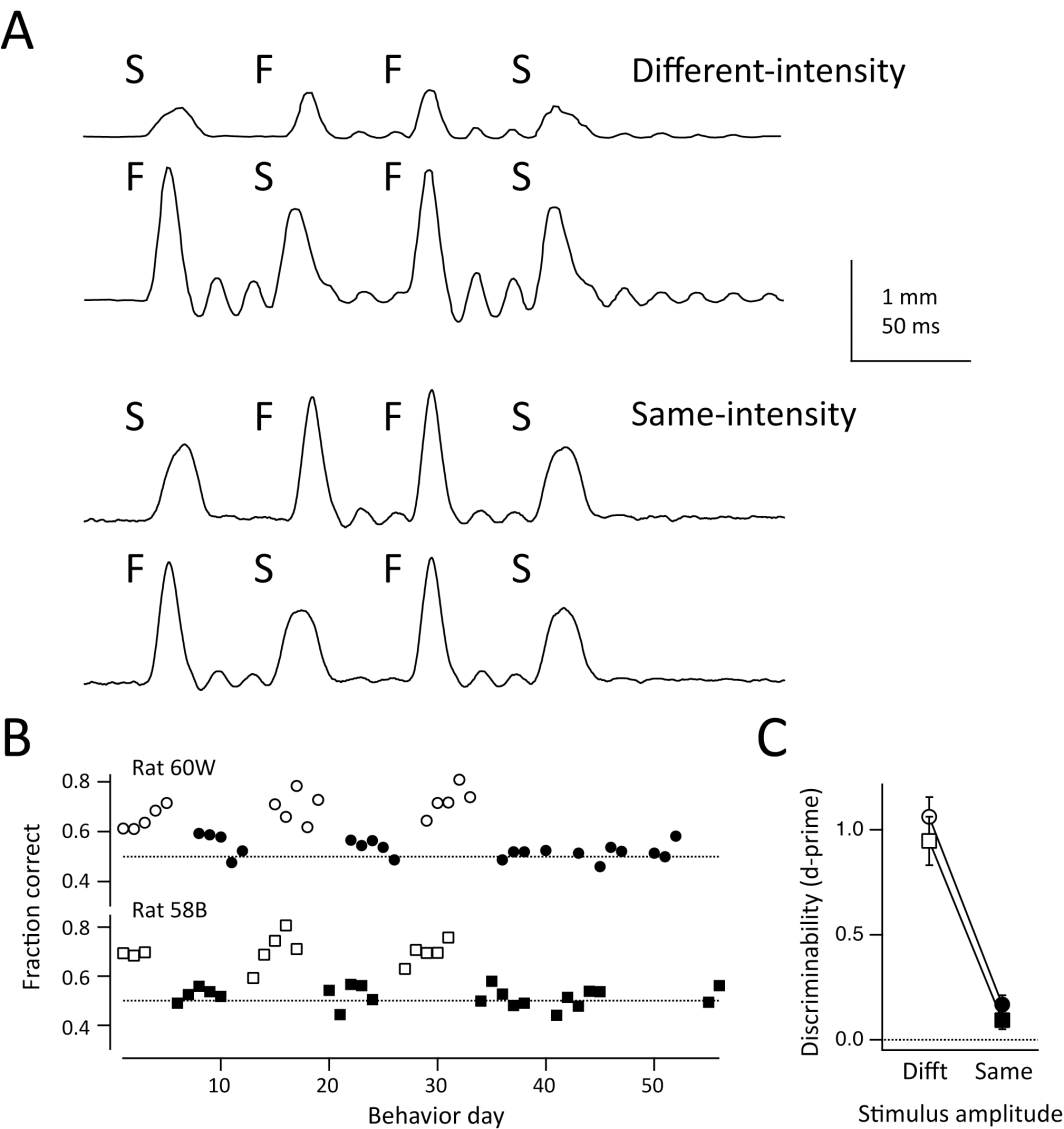
Behavioral performance on FSFS-SFFS discrimination task. (A) Panel kinematics for SFFS and FSFS sequences, showing both different-intensity and same-intensity versions. (B) Behavioral performance across all behavioral sessions, for the two rats trained on this task. Open symbols: sessions using the different-intensity version of the stimuli. Filled symbols: the same-intensity version. Both rats could discriminate the different-intensity version but not the same-intensity version. (C) D-prime analysis of discrimination performance for the same two rats (circles: 60W, squares: 58B), across all behavioral sessions. Symbols are mean ± SEM across sessions. Data for this figure are in S1 Data.

To test whether failure to discriminate FMS versus SMF reflected insufficient training on these sequences or the presence of easier FFF and SSS stimuli on 50% of trials, we trained two rats on a modified task. This used a very simple task structure with only two stimuli: an FSFS sequence (rewarded at the right drink port) and an SFFS sequence (rewarded at the left drink port). F and S impulses had 216 and 120 mm/s peak velocity and 1.2 and 0.7 mm amplitude, respectively. Both trains had 34 ms interpulse interval and 188 ms total duration (Fig 3A). We constructed two sets of stimuli: a “same-intensity” version in which FSFS and SFFS trains had nearly identical mean speed (25.7 and 26.4 mm/s, calculated across the full sequence), and a “different-intensity” version in which FSFS and SFFS stimuli were scaled in amplitude so that mean speed was 27.8 and 8.7 mm/s, respectively.

Two rats (58B and 60W) were initially trained to discriminate the different-intensity sequences (>65% correct over 3 d). Then, we replaced these stimuli with the same-intensity FSFS and SFFS sequences, so that discrimination could only occur by detecting differences in fine temporal structure, not mean speed. Performance dropped to chance and did not improve over 5 d of training (Fig 3B). We then alternated weekly training on different- and same-intensity sequences. Both rats consistently discriminated FSFS from SFFS when they had different mean speed (58B: 70 ± 1.5% correct, 60W: 69.2 ± 1.6%), but not when they had the same mean speed, even after >20 cumulative days of training (58B: 52 ± 0.8% correct; 60W: 53 ± 0.8% correct). This was evident in the d-prime measure of discrimination between FSFS and SFSF stimuli, which was 1.02 for different-intensity stimuli and 0.12 for same-intensity stimuli (Fig 3C). Thus, behavior correlated with the presence of a slow, integrated cue.

### S1 Recordings during Behavioral Discrimination

**Fig 4:**
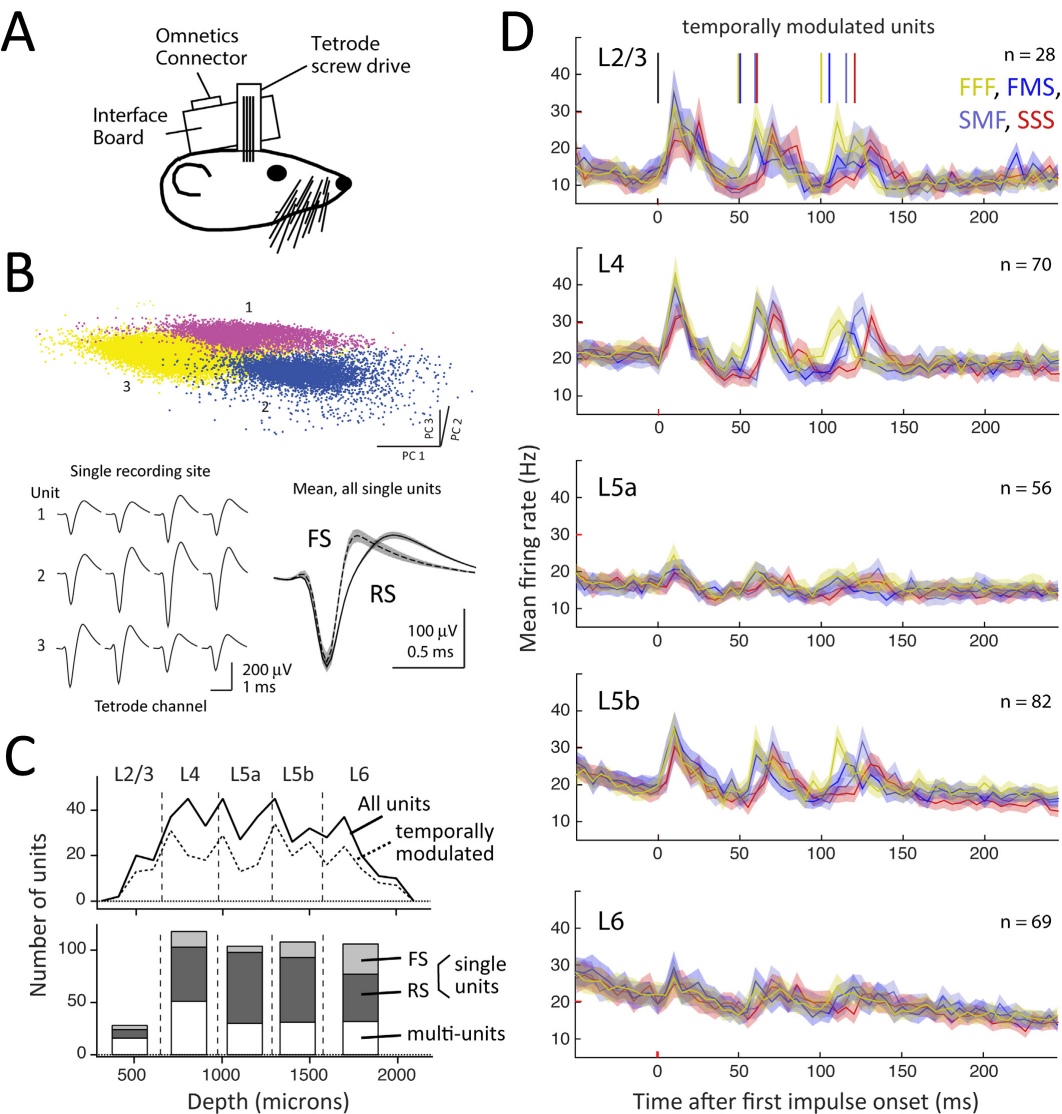
S1 recordings during discrimination behavior. (A) Schematic of multi-tetrode chronic microdrive. (B) Cluster separation for one recording site (top) with mean spike waveforms for three simultaneously recorded single units (bottom left). Bottom right, mean spike waveform for all fast-spike (FS) and regular-spike (RS) single units. (C) Laminar distribution of recorded units. (D) Population peri-stimulus time histogram (PSTH) for all temporally modulated units by layer and stimulus type. Different sequences have different onset times for impulses 2 and 3 (colored ticks). Data for this figure are at crcns.org repository (accession ssc-4).

To study S1 coding of whisker sequences during vibrotactile discrimination, we recorded S1 spiking during the FFF-FMS-SMF-SSS behavioral task using chronic multi-tetrode microdrives. Four tetrodes (~350 um lateral spacing) were driven as a group, enabling simultaneous recording of many neurons in several whisker-related columns (Fig 4A). Tetrodes were initially implanted into mid-L2/3 and advanced by ~140 μm every one to two recording sessions, sampling neurons from L3 to L6 over 12–22 d of recording. Spike sorting yielded 3.8 (range: 0–11) well-separated single units per recording session (Fig 4B). Additional units showed clear separation from noise but failed the interspike interval criterion for single units and were classified as multi-units. We obtained 306 single units and 167 multi-unit clusters (total: 473 units) across 80 recording sessions in five rats (18FB, 18Ri, 18Ro, 62BS, 107St), spanning across L3 to L6 (Fig 4C). Fast-spike (FS) and regular-spike (RS) units were well separated by spike width. Recordings were localized to C1-4, D2-4, and E3 columns based on receptive field mapping under light isoflurane anesthesia and recovery of marking lesions. These whiskers were visually confirmed to contact the panel, as in a prior study using this behavioral apparatus [22].

Mean firing rate during a 25-ms prestimulus baseline period in the nose poke was 6–10 Hz across layers for RS units, 8–32 Hz for FS units, and higher for multi-unit clusters (S1 Table). Lowest firing rates were observed in L2/3, L4, and L6. Firing rate distributions were positively skewed (S3A Fig). Firing rates for RS units were higher than in prior studies using cell-attached or whole-cell recording in rodents whisking mostly in air [6,24,25]. This likely reflects recording bias for more active units and the fact that whiskers contacted the stimulus panel through the entire nose poke duration, including the baseline period.

We first identified units whose average firing rate was significantly temporally modulated with any dynamics during the nose poke period (*p* < 0.05, temporal modulation permutation test, see Materials and Methods). Three hundred five out of 473 units (63.5%) showed significant temporal modulation. Temporally modulated units were distributed uniformly across whisker columns and layers (Fig 4C) and had higher baseline firing rates than non-modulated units (S3B Fig). Subsequent analysis focused only on these temporally modulated (i.e., task-involved) units. Single- and multi-units showed similar response properties and were combined for analysis unless indicated.

The average population response, compiled across all temporally responsive units in each layer, was dominated by a brief, phasic increase in firing rate following each panel impulse (Fig 4D). This was greatest in L2/3, L4, and L5b, and weakest in L5a and L6. The mean impulse-evoked firing rate modulation (in Hz above pre-impulse baseline) was 14.2 ± 2.3 in L2/3, 15.2 ± 1.9 in L4, 6.3 ± 1.2 in L5a, 14.4 ± 2.3 in L5b, and 7.0 ± 1.5 in L6 (*n* = 28–82 units per layer). Among units with significant impulse responses, peak response latency was shortest in L4, L5a, and L5b (9.8, 10.3, and 12.0 ms) and longest in L2/3 and L6 (13.8 and 16.1 ms). Superimposed on these phasic responses to individual impulses was a gradual decrease in average firing rate during the nose poke period, observed in all layers except L5a (Fig 4D).

**Fig 5:**
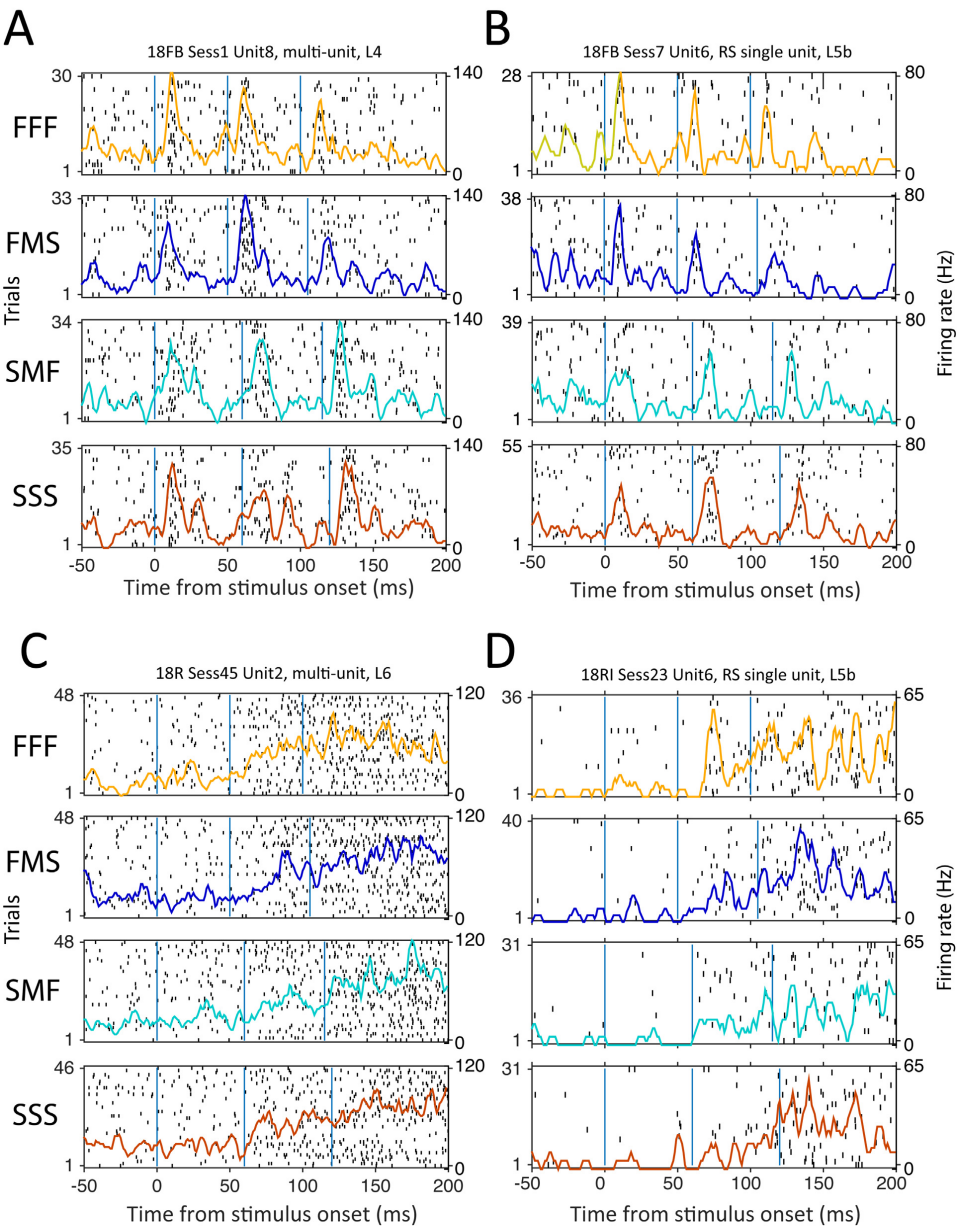
Sequence responses for example units. (A and B) L4 multi-unit and L5b RS single unit with phasic response to each impulse. (C and D) L6 multi-unit and L5b RS single unit with increasing firing rate during the stimulus period. Each panel shows the spike raster and PSTH across trials, for one stimulus sequence. Vertical lines: onset of each impulse. Data for this figure are at crcns.org repository (accession ssc-4).

Individual units most commonly showed phasic responses to individual impulses (examples, Fig 5A and 5B). However, some units instead showed cumulatively increasing firing rate during the stimulus period (Fig 5C and 5D) or decreasing firing rate (not shown). These were intermixed in the same columns and recording sites.

### Regression Analysis to Identify Fast- and Slow-Time Scale Units

**Fig 6:**
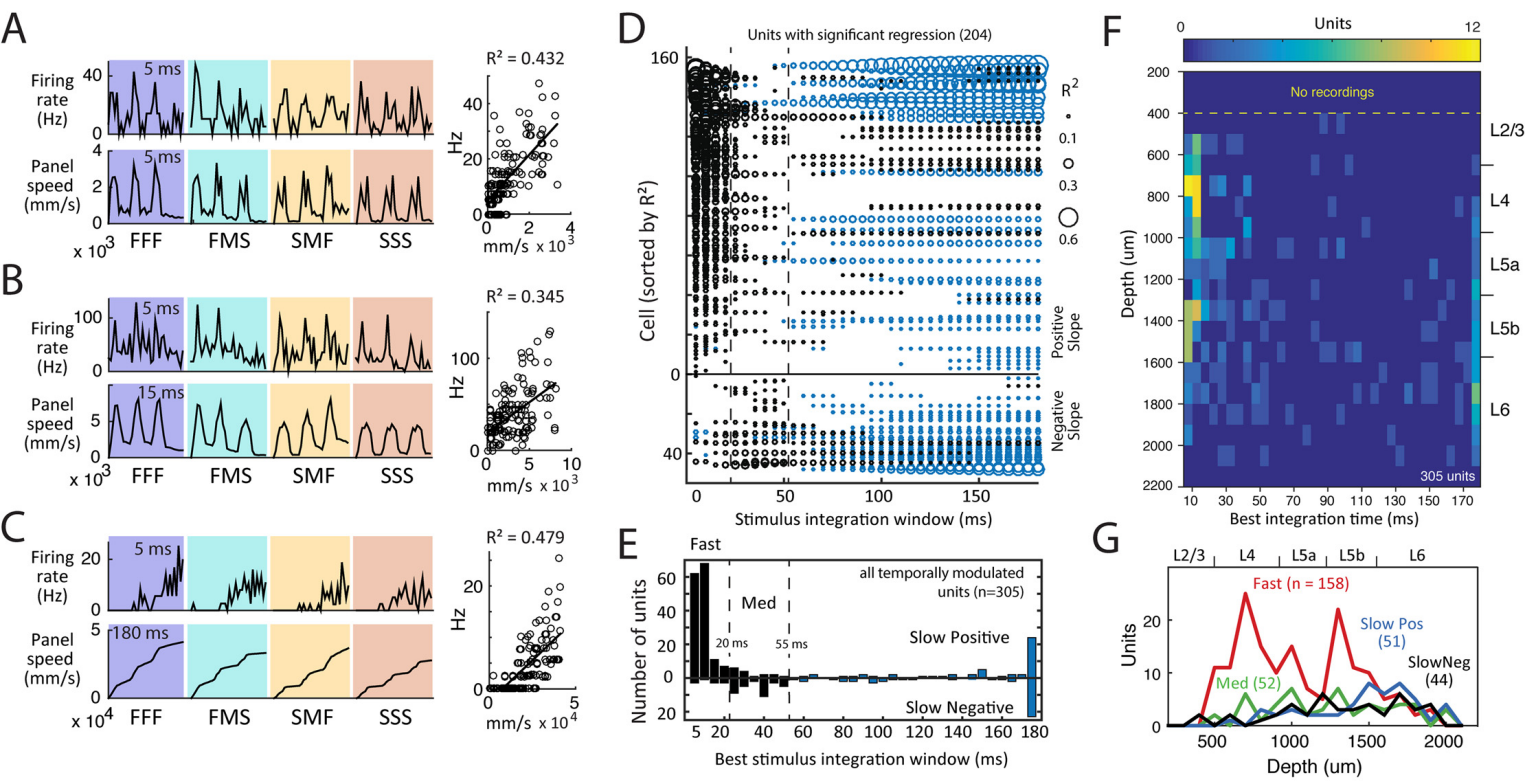
Classification of S1 units by stimulus regression. (A–C) Stimulus regression for three example units. Top, PSTH in 5 ms time bins. Bottom, stimulus panel speed integrated over 5, 15, or 180 ms, which was the best fit stimulus integration window for each unit. Right, regression of firing rate on integrated stimulus speed. (D) Coefficient of determination (R^2^) for all stimulus integration windows with a significant regression, for each unit with a significant regression to at least one window. Black: Fast and Medium time scale units (best integration window <55 ms). Blue: Slow units. Cells are sorted by peak R^2^ and by sign of the regression slope for the best integration window. (E) Number of units with each best integration window and positive or negative regression slope. (F,G) Laminar distribution of units by best integration window. Data for this figure are in S1 Data.

To quantify the time scales of stimulus representation in S1, we performed a multiple regression analysis for each temporally modulated unit (*n* = 305), whose goal was to identify the time window of stimulus integration that best predicted the neuron’s firing rate (Fig 6). The dependent variable was firing rate, in 5 ms bins, calculated over all trials for each stimulus sequence. The regressors were integrated speed of panel movement over a variety of temporal integration windows (5, 10, 15, … 180 ms, for a total of 36 regressions). Firing rate in each 5 ms bin was predicted from the integrated panel speed in the preceding bin. Two hundred four units showed a significant regression for at least one stimulus integration window (α = 0.05/36 = 0.0014, using Bonferroni correction for the multiple regressions). For each unit, we defined the best fit integration window as the stimulus integration window with the highest R^2^ value.

Most units had a short best fit integration window (5–20 ms), indicating that firing rate was best predicted by stimulus speed on a short time scale (examples, Fig 6A and 6B). However, some units exhibited slowly increasing or decreasing firing that was correlated with integrated speed over long timescales, most often the whole stimulus period (example, Fig 6C). Individual cells had high R^2^ values for either short or long integration windows but rarely both (Fig 6D). Most units showed a positive regression slope for the best integration window, indicating that firing rate increased with integrated stimulus speed, while ~20% showed a negative slope (Fig 6D and 6E). Empirically, units with 5–20 ms best integration windows (Fast units; *n* = 158) had positive slopes. Units with 25–55 ms integration windows were rarer (Medium units; *n* = 52) and had largely negative slopes. Units with slow (55–180 ms) integration windows had either positive regression slope (Slow Positive units; *n* = 51) or negative regression slope (Slow Negative units; *n* = 44).

Fast units were 73% of temporally modulated units in L2/3 and L4, 50% in L5, and 23% in L6. Overall, 52% of temporally modulated units were Fast units. Both Fast and Medium units were most prevalent in L2/3, L4, and L5b. In contrast, both Slow Positive and Slow Negative units were located primarily in L5 and L6 (Fig 6F and 6G). Overall, slow units were 13% of temporally responsive units in L2/3 and L4, 31% in L5 and 56% in L6. Fast, Medium, Slow Positive, and Slow Negative categories each contained both single- and multi-units and both RS and FS units.

### Fast and Medium Time Scale Units

**Fig 7:**
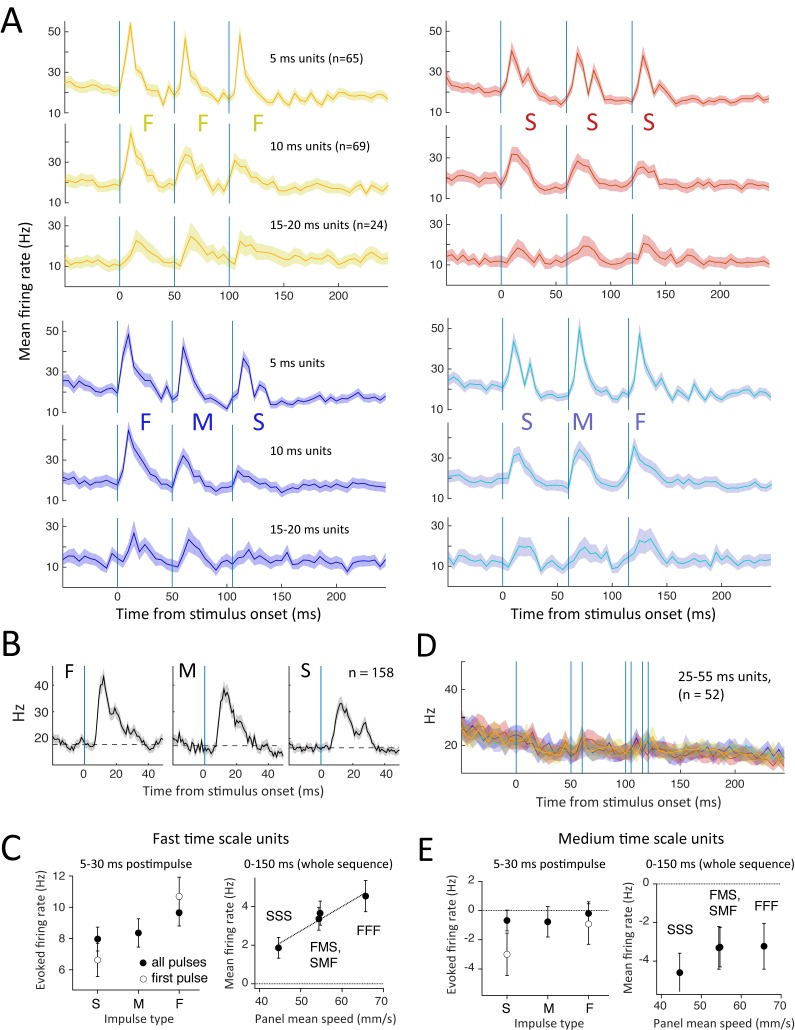
Stimulus coding by fast time scale units. (A) Population PSTH (mean ± SEM) for Fast units with 5, 10, and 15–20 ms best integration windows. (B) Population PSTH for all individual F, M, or S impulses, irrespective of sequence membership, for all Fast units. Dashed line: pre-impulse firing rate. (C) Left: net evoked rate for individual impulses, calculated as post-impulse rate–pre-impulse rate. Right: mean rate across the entire sequence above pre-stimulus baseline, as a function of mean panel speed. Symbols show mean ± SEM across units. Line: regression. (D) Population PSTH for Medium units for FFF, FMS, SMF, and SSS sequences. (E) Net evoked rate for individual impulses and mean rate across the sequence for Medium units. Conventions as in C. Firing rate was suppressed by all impulses and sequences. Data for this figure are at crcns.org repository (accession ssc-4).

Fast time scale units showed temporally precise coding of individual panel impulses and sequences (Fig 7A–7C). Population peri-stimulus time histograms (PSTHs) for the fastest units (5 ms best integration window) showed responses to F impulses (16 ms duration) that lasted just ~20 ms and responses to S impulses that tracked impulse onset and offset separately. Units with 10 ms and 15–20 ms best integration windows had somewhat slower responses, as expected, but still tracked individual impulses. Adaptation within each train was quantified as mean firing rate to pulse N/pulse 1 and was modest in FFF trains (2/1: 0.80 ± 0.11, 3/1: 0.70 ± 0.14, *p* < 0.05 by *t* test, *n* = 61 single RS units with significant response to F impulses) and statistically absent in SSS trains (2/1: 1.09 ± 0.26, 3/1: 0.86 ± 0.35, all mean ± SEM) (Figs 7A and S4). This is less adaptation than reported for non-whisking, non-task-engaged rats [16,26] and is similar to passive whisker detection [10].

To determine whether Fast units accurately discriminate impulse velocity, we calculated the average response to all individual F, M, or S impulses (compiled across all sequences). The firing rate of Fast units (*n* = 158) in a brief window after each impulse was greater for F versus S impulses, and intermediate for M impulses (Fig 7C, left). Mean firing rate measured over the entire duration of a sequence (0–150 ms after sequence onset) varied closely with mean speed of the sequence, being highest for FFF, lowest for SSS, and intermediate and equal for FMS and SMF (Fig 7C, right). Thus, population average firing rate of Fast units over the entire sequence closely matched the mean behavioral performance of the animals (Fig 2C).

In addition to coding pulse velocity, Fast unit coding was also influenced by pulse order because of adaptation. Fast RS single units (*n* = 61) showed greater adaptation during FFF than SSS sequences. Consistent with this, the middle M pulse in FMS sequences appeared weaker than in SMF sequences, though this did not achieve statistical significance (*p* = 0.08, paired *t* test, *n* = 61 units) (S4A Fig). Thus, Fast units represent impulse velocity, but with some history dependence due to adaptation, and no sign of positive temporal integration across impulses.

In contrast, medium time scale units responded to impulses with a modest decrease in firing rate, rather than an increase, consistent with the negative regression slope for most of these cells (Figs 6E and 7D). In firing rate analysis, these cells were inhibited by F, M, and S impulses and did not distinguish either individual impulse identity or whole sequence identity (Fig 7D and 7E). Thus, medium time scale units do not represent stimulus information useful for this discrimination task.

### Slow Positive and Slow Negative Units

**Fig 8:**
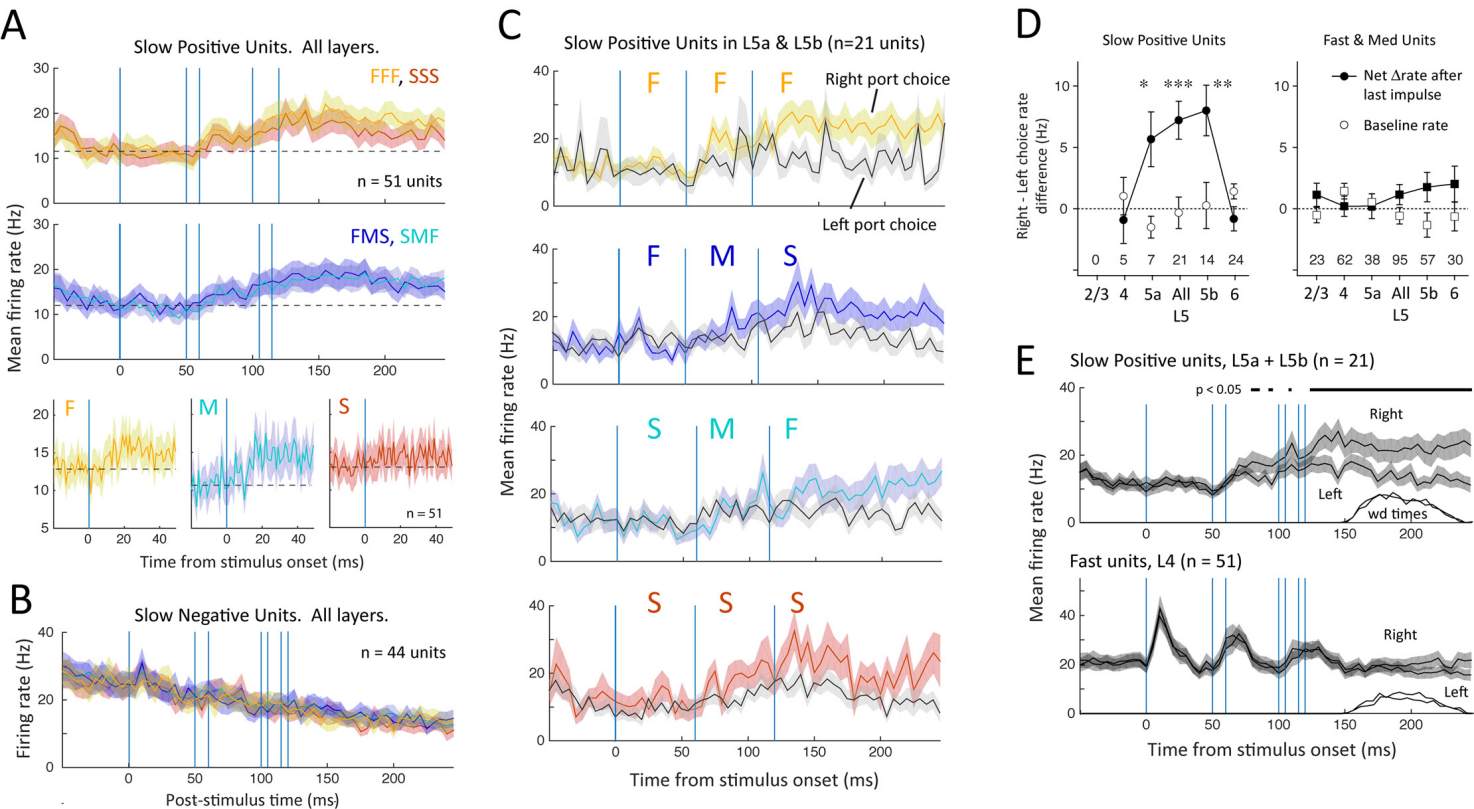
Choice coding by slow time scale units. (A) Top: population PSTH (mean ± SEM) for Slow Positive units across all layers. Responses were indistinguishable between FFF, FMS, SMF and SSS trains. Bottom: population PSTH for individual F, M, and S impulses, irrespective of sequence membership. (B) Population PSTH for slow negative units, showing lack of any impulse-evoked firing rate modulation. (C) Population PSTH for slow positive units in L5a and L5b, separated by stimulus type and drink port choice. Slow Positive units fired more on right-choice trials for all stimuli. (D) Difference in evoked rate between right- and left-choice trials, measured 5–50 ms after start of the final impulse, for all Slow Positive units (left) or Fast and Medium units (right). Number of units in each layer is shown at bottom. Open symbols, baseline rate before sequence onset for the same trials. * *p* = 0.022; ** *p* = 9.5 x 10^-4^; *** *p* = 7.5 x 10^-5^, paired *t* test comparing rate on right versus left choice trials. (E) Population PSTH averaged across all four sequences, for right- versus left-choice trials, for Slow Positive units in L5 (top), and for Fast units in L4 (bottom). Bar shows times when rate is significantly different between right- and left-choice trials by sliding *t* test (*p* < 0.05). The distribution of nose poke withdrawal times is shown for the same trials. Data for this figure are at crcns.org repository (accession ssc-4).

Slow positive units (*n* = 51) also showed a time-locked increase in firing rate after panel impulses, on average, but mostly to the second and third impulses in the sequence. Responses were small and sustained (unlike the large, transient responses by Fast units) and were evident for F and M impulses but not S impulses (Fig 8A). However, mean firing was not different for FFF, FMS, SMF, or SSS trains, suggesting that these neurons do not appreciably integrate impulse information for sequence discrimination (Fig 8A). Slow negative units did not respond to impulses at all, and firing rate steadily declined over time, not locked to panel impulses (Fig 8B).

Unexpectedly, firing of Slow Positive units correlated with the animal’s behavioral choice on each trial. Fig 8C shows population PSTHs for Slow Positive units in L5a and L5b, divided into trials in which the rat chose the right- or left-side drink port. Slow Positive units fired more on trials when the rat chose right (contralateral to the S1 recording). This was true for both FFF and FMS stimuli, for which right was the correct response, and SMF and SSS stimuli, for which right was the incorrect response. We quantified right-choice bias as the firing rate difference on right versus left trials, measured 5–50 ms after the start of the final impulse. Right-choice bias was significant for Slow Positive units in L5a and L5b, but not other layers (Fig 8D). Firing rate began to diverge on right versus left choice trials after the second impulse and was consistently significant by 125 ms, which is during the third impulse (*p* < 0.05, sliding paired *t* test) (Fig 8E). This preceded the earliest withdrawals (150 ms) and mean withdrawal time (190 ms). Choice-related activity was absent in fast time scale units in L4 (Fig 8E).

Thus, L5 Slow Positive units exhibited weak impulse-evoked spiking and strong choice-related spiking (Fig 8). We tested for stimulus integration in these units by comparing firing rate during each impulse of FFF, FMS, SMF, and SSS sequences on right- and left-choice trials separately, which removes choice as a factor (S5 Fig). Evoked firing was minimal for pulses 1 and 2 and was not correlated with pulse velocity. Pulse 3 firing rate was higher but was essentially identical for FFF, FMS, SMF, and SSS sequences and did not correlate with mean speed of the entire sequence or of the last two impulses. Thus, these units did not effectively summate stimulus information across impulses.

We asked whether choice-related firing could reflect a feed-forward sensory reafferent signal generated by decision-related movements in the nose poke. Reafference from fast whisker deflections is unlikely, because L4 Fast units did not exhibit choice-related firing (Fig 8). However, a distinct slow reafferent signal is possible. We tested for choice-related postural movements by analyzing high-speed videos in 43 trials (22 left choice, 21 right choice) from four rats. In each trial, we tracked head position, head angle, and whisker tip position with ~100 μm precision at 8.4-ms intervals from 0 to 150 ms after stimulus onset. Head angle and whisker tip trajectories were invariant between right- and left-choice trials. Head position differed modestly between right- and left-choice trials beginning at 100 ms, with a 0.6 mm difference at 125 ms (S6 Fig). Thus, slow head movements are a potential reafferent driver of choice-related firing in L5.

### RS and FS Single Units

Fast, Medium, Slow Positive, and Slow Negative response classes all included RS, FS, and multi-unit clusters, although few FS cells were found in the Slow classes (S7 Fig). Among Fast units, all three unit types had similar sequence-related PSTHs. Among L5 Slow Positive units, both RS units and multi-unit clusters had similar choice-related firing, and no FS units existed in this category (S7 Fig). Thus, all response classes involved RS units.

### Neural Decoding of Stimulus Identity and Behavioral Choice

**Fig 9:**
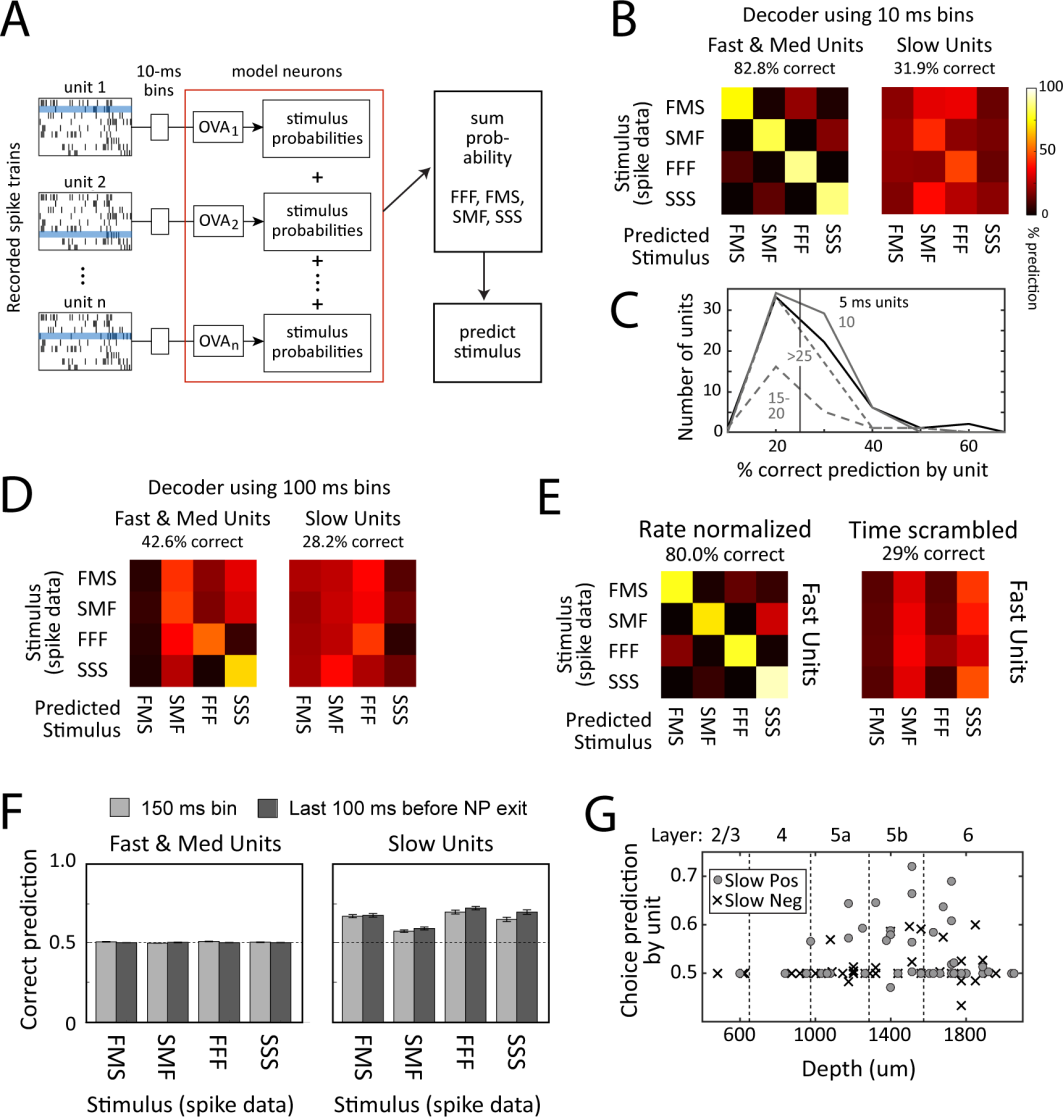
Population decoding of sequence identity and behavioral choice. (A) Decoder architecture for stimulus decoding. Each neuron was represented by a one-versus-all (OVA) classifier, trained by logistic regression to calculate the probability of each stimulus given a single-trial spike train (blue trial). The stimulus with the highest summed probability across neurons was taken as the population prediction. (B) Average performance of stimulus decoder with 10 ms time bins, constructed from all Fast and Medium units or all Slow units. The title reports average percent correct classification across all four stimuli. Entries along the diagonal are percent correct, and rows sum to 1.0. (C) Percent correct performance for each unit in the Fast/Medium model in (B), separated by best integration window. Vertical line, chance prediction of 25%. (D) Average performance of stimulus decoder with a single 150 ms time bin. (E) Average performance of a Fast/Medium stimulus decoder with 10-ms bins, using rate-normalized or time-scrambled spike trains. (F) Performance of behavioral choice decoders, built from Fast and Medium units or Slow units, using two different bin sizes. Chance performance is 50%. (G) Choice prediction for each unit in the Slow model, using a single 100-ms bin prior to nose poke withdrawal. Units are separated by depth and response type. Best choice prediction was by Slow Positive units in L5b. Data for B–E are in S1 Data.

S1 neurons spike sparsely, with individual whisker deflections eliciting mostly zero spikes, occasionally one spike, and, very infrequently, two spikes on a single trial [21,27,28]. We also observed this highly variable, sparse single-trial spiking behavior (Fig 5). To test whether S1 accurately encodes whisker sequences on single trials, we constructed a neural population decoder that predicted stimulus identity from single-trial spike trains. In the model, each recorded neuron was represented by a separate, independent one-vs-all (OVA) classifier that predicted the probability of each sequence (FFF, FMS, SMF, or SSS) given one spike train, chosen randomly from that neuron’s recorded spike trains in vivo, and binned in discrete time bins. Each OVA classifier was trained by logistic regression from a randomly chosen subset of spike trains for that unit. The output of each classifier was the probability of each stimulus type versus all others, based on the presented spike train. To create a population prediction, stimulus probabilities were summed across units, and the sequence with highest summed probability was taken as the population stimulus prediction (Fig 9A). This model assumes independence between neurons and allows stimulus prediction by both firing rate and temporal information within spike trains.

We first constructed a decoder from all Fast and Medium units, using 10 ms time bins. This model predicted sequence identity, using one single-trial spike train per model unit, with 83% overall accuracy (range: 74% for FMS to 88% for FFF spike trains). Chance performance is 25% (Fig 9B). The individual neurons with best stimulus prediction were those with 5–10 ms best integration windows (Fig 9C). Remarkably, this model identified SMF and FMS sequences with 78% accuracy, even though rats could not. A second decoder constructed of all Slow units, also using 10 ms bins, predicted sequence identity at near chance levels (32% correct, not significantly different from chance, *p* = 0.47) (Fig 9B). Decoding from mean firing rate in a single 150-ms bin substantially reduced Fast/Medium decoder accuracy (43% correct) and did not improve Slow decoder accuracy (Fig 9D).

To test whether the Fast/Medium model recognized sequences by mean firing rate or temporal spike pattern, we rate-normalized the spike train data (preserving temporal information across the 10-ms bins) or time-scrambled spike trains within trials (preserving firing rate information). Fast/Medium decoders trained on rate-normalized data performed well (80% correct), but time-scrambling spikes abolished performance (Fig 9E). Thus, the Fast/Medium decoder primarily identified stimuli by temporal spike patterns, which varied between FFF, FMS, SMF, and SSS sequences (Fig 7). Thus, sequence identity was primarily encoded in short time-scale spiking information, carried by Fast units.

We constructed a similar decoder to predict behavioral choice. This was trained on spike data from all four sequences and was tested for prediction of right versus left drink port choice separately for FFF, FMS, SMF, and SSS trials. A choice decoder based on Fast/Medium units was unable to predict drink port choice, either using 10 ms bins (not shown), mean firing rate in a single 150-ms bin, or mean firing rate in the last 100 ms prior to nose poke withdrawal (Fig 9F). A choice decoder based on Slow units successfully predicted drink port choice using a single 150-ms bin, or mean firing rate in the last 100 ms before nose poke withdrawal (65% correct for both models) (Fig 9F). Post-hoc analysis showed that units with best choice prediction were Slow Positive units located primarily in L5b (Fig 9G). Thus, spiking of Slow Positive units was sufficient to decode behavioral choice but not sequence identity.

## Discussion

### Behavioral Integration of Stimulus Sequences

Cortical sensory systems temporally integrate sensory signals for many types of perceptual decision-making [2]. Where and how integration is performed is unclear. In fingertip vibrotactile discrimination by primates, S1 neurons spike to each rapid skin deflection, and this information is temporally integrated downstream of S1 to guide behavioral discrimination [1,29]. In the rodent whisker system, passive vibrotactile discrimination is often based on slow, time-integrated input [12,13], although rats are also capable of discrimination based on rapid kinematics [8]. Integration is also implicated in discrimination of surface texture (roughness), in which surface whisking generates temporally dense sequences of stick-slip whisker micromotions, whose mean statistics, including mean whisker speed, correlate with roughness [3,4,21,30–33]. S1 neurons spike phasically to stick/slip events and other features such as dynamic changes in whisker bend [3,21,34], and behavioral judgments of surface roughness correlate with mean firing rate and rate of synchronous spiking across S1 neurons [21,35,36]. Thus, roughness discrimination likely involves temporal integration of stick/slip events and S1 spike trains. Integration is useful because it reduces the complexity of the vibrotactile signal to a single scalar quantity of stimulus intensity. Intensity-based discrimination is common across modalities and is a defining feature of texture discrimination in people and non-human primates [37]. Integration is also evident in whisker-based object localization, in which S1 spikes are time-locked to object contact, but mice judge object location by behaviorally integrating spike counts over ~50 ms, rather than using precise timing [19].

In our task, rats were able to distinguish FFF versus SSS sequences that differed in mean speed, but not FMS versus SMF sequences that had the same mean speed, and choice behavior was strongly related to mean speed across the sequence (Fig 2). Similar performance was observed in the SFSF versus FSSF task (Fig 3). Task performance was relatively low (d-prime for FFF versus SSS: 0.5–1.5), as in a prior study [13], indicating the difficulty of these tasks. The results suggest that rats utilized slow, time-integrated information for task performance, even though simple, short time-scale cues (e.g., identity of the first impulse) would have led to more rewards. This hypothesis is consistent with two prior vibrotactile discrimination studies using a similar design, in which rapid kinematics and slow intensity cues were manipulated separately to prove that rats guided discrimination by slow, time-integrated cues [12,13]. We did not test this causally in our study, so we cannot rule out that rats may have solved our task using a hidden short time scale cue.

Integration is not required for simpler detection tasks [7,9,10] or detection-of-change tasks [8], and rodents can perceive single brief whisker impulses within ongoing deflection trains [7–11,38]. This suggests that rats generate neural codes for both rapid and integrated features that guide different aspects of sensory-guided behavior. Rats may differentially use these codes depending on task demands and training strategies. In our task, initial training involved strong intensity cues, which may have promoted adoption of an integration-based strategy. An intensity-like feature of vibrotactile stimuli is encoded in primate dorsolateral prefrontal cortex during a working memory task [39], but no explicit intensity representation is known yet in the rodent whisker system.

### Stimulus Encoding in S1 Occurs at Fast (5–20 ms) Time Scales

We tested for stimulus integration in S1 during task performance but found that S1 encoded whisker sequences almost exclusively at very rapid time scales. Forty-four percent and 52% of temporally responsive units showed very fast (5–10 ms) and fast (5–20 ms) stimulus integration, respectively (Fig 6E). These units spiked to individual whisker impulses, with firing rate encoding impulse velocity, and mean firing rate correlated with mean whisker speed across the sequence (Fig 7A–7C). Seventeen percent of units showed firing rate modulations on medium (25–55 ms) time scales, but these were inhibited by whisker impulses and did not discriminate different impulses or sequences (Fig 7D and 7E). Sequence identity could be decoded accurately from Fast units but not Medium units, and stimulus information was abolished by scrambling spike times across 10-ms bins. Thus, Fast units encode sequence identity by representing the velocity and timing of individual impulses. Fast units accurately distinguished FMS from SMF sequences, even though rats could not (Fig 9B). Thus, accurate short time-scale representation of vibrotactile sequences exists in S1 but does not appear to be used efficiently to guide behavior in our task. This is identical to primate S1, in which precise spike timing discriminates vibrotactile flutter more accurately than the animal [40].

Fast units had phasic whisker responses similar to classic anesthetized studies [14,41] and S1 units recorded during detection tasks [7,8,11]. Responses were weak in L5a and L6 (Fig 4), which may reflect involvement of this layer in active whisking, which was absent in our task [42]. Adaptation was minimal: ~25% for FFF trains and absent for SSS trains (Fig 7A–7C). This level of adaptation is less than occurs under anesthesia [15,18] or in quiescent, non-task engaged rats [16,26] and is similar to that during active exploration [16,26] or in a whisker detection task [10]. While adaptation generates history dependence and thus carries information about prior impulses [43,44], Fast units showed no evidence of positive integration across impulses.

### Slow Units Do Not Integrate Stimuli but Reflect Behavioral Choice

Seventeen percent of units, primarily in deep layers, were Slow Positive units with small, sustained responses to individual whisker impulses and progressively increasing firing rate during the stimulus period. However, these units did not accurately encode or integrate whisker impulses. Responses were generally absent to the first impulse of sequences, and firing rate did not differ between FFF, FMS, SMF, and SSS sequences or correlate with mean speed (Figs 8A and S5). Thus, Slow Positive units do not appear to carry integrated stimulus information for sequence discrimination. Slow Negative units had slowly decreasing firing rate and no stimulus-related firing modulation at all (Fig 8). Consistent with these observations, sequence identity could not be decoded from Slow unit spike trains (Fig 9B). Slow whisker-evoked spiking occurs in some L2/3 units in mice [20] but was not evident in our dataset in rats.

Instead, firing of Slow Positive units in L5 was strongly related to drink port choice. Choice-related spiking [45] occurs in many cortical areas, including primary visual cortex [46], S1 of primates and rodents [11,47–49], and even subcortically [49,50]. In rodent S1, many L2/3 neurons exhibit choice-related spiking in near-threshold detection tasks [11,49]. Choice-related firing emerged significantly after the second impulse of the sequence and was consistent during the third impulse, 65 ms before the average nose poke withdrawal (Fig 8E). A neural decoder built from Slow unit spike trains predicted behavioral choice from mean firing rate in the stimulus period and in the 100 ms prior to nose poke withdrawal (Fig 9). Choice-related firing was absent in L4 Fast units, suggesting it did not represent reafference from fast whisker sensory signals (Fig 8E). Choice-related spiking could reflect reafference from slow head movements prior to nose poke withdrawal, potentially mediated by POm afferents to L5 [51] or an internal decision or motor preparatory signal. Its onset after the second impulse could reflect an early behavioral decision based on first and second impulse stimulus information or an early stimulus-independent “"guess” that biased subsequent stimulus-dependent drink port choice. Thus, Slow Positive units do not appear to integrate across whisker impulses but combine weak impulse responses with a distinct, slow signal related to behavioral choice.

### Where in the Brain Does Temporal Integration Occur?

We found that during vibrotactile discrimination, most S1 neurons represent the velocity and timing of individual whisker impulses at rapid, 5–20 ms time scales. While there was some history dependence of whisker responses due to modest adaptation, we did not observe evidence of positive integration across whisker impulses in S1 firing rates. Thus, temporal integration for discrimination is likely to occur downstream of S1, in higher sensory or premotor regions. These may include S2, prefrontal cortex, and premotor cortex, as in primate vibrotactile discrimination [1]. We cannot rule out that S1 could learn to temporally integrate under conditions in which rats were more reliant on slow cues for behavioral discrimination. For whisker texture perception, our finding of short time scale coding in S1 suggests that S1 primarily encodes low-level kinematics of individual stick/slips and bends [6,21], which are integrated downstream to represent texture or other surface features.

## Materials and Methods

Female Long-Evans rats were >3 mo of age. All procedures were approved by the UC Berkeley Animal Care and Use Committee (protocol R309-0516BC) and comply with NIH guidelines.

### FFF-SMF-FMS-SSS Discrimination Task

The computer-automated chamber contained a nose poke, flanked by a wall-mounted whisker stimulus panel (2 x 2 cm) that was carried on a hidden piezoelectric actuator (Piezo Systems PSI-5H4E). Whiskers were trimmed to 15 mm in length. The right-side C, D, and E row whisker tips rested against the panel while the rat was in the nose poke (Fig 1A). Nearby right and left drink ports contained infrared-LED beam sensors to detect nose entry and delivered calibrated water rewards. Trials were monitored by infrared video.

Each trial was self-initiated by entry into the nose poke. After a variable delay (75–100 ms), a sequence of three rapid whisker deflections was delivered via the panel. The rat was required to remain in the nose poke for 250 ms to ensure full sequence delivery. The rat then withdrew from the nose poke and was rewarded (0.05–0.1 mL water) for choosing the drink port that was associated with the presented stimulus. Incorrect drink port choice or premature nose poke withdrawal triggered a time-out tone (4–6 s) and no reward. In a subset of sessions, high-speed video (119 Hz) was recorded.

#### Whisker sequences

Each whisker deflection sequence consisted of three up-down ramp-return deflections (pulses). Each pulse had either slow (S), medium (M), or fast (F) rise-fall velocity. These pulses differed in rise-fall time and therefore had different pulse durations but similar amplitude (Fig 1B and 1C; Table 1). Sequences had either FFF, FMS, SMF, or SSS pulse order, with 34 ms between the end of one pulse and the beginning of the next, yielding 50–62 ms interval between pulse onsets. Total train duration (from beginning of the first pulse to end of the last pulse) was 120–146 ms. Mean speed, calculated over the entire train, was highest for FFF, intermediate and equal for FMS and SMF, and lowest for SSS sequences (Fig 1D; Table 1; S1 Fig). One sequence was presented per trial, with random order across trials. Training was in the dark, and acoustic cues were obscured using masking noise composed of white noise densely intermixed with sampled piezo sounds. To further mask any unintended auditory cues, an additional “dummy” piezo was hidden behind the stimulus panel and actuated on each trial in a manner uncorrelated with panel movement.

#### Training stages and reward contingency

First, rats were trained to nose poke for >150 ms and to drink from the drink ports. Next, rats were presented in the nose poke with exaggerated amplitude and velocity versions of FFF and SSS stimuli and were trained to choose the right drink port for FFF stimuli and the left drink port for SSS. When each rat achieved >60% correct, stimulus amplitude was stepped closer to the final amplitude, and the nose poke time requirement was incrementally increased. This was iterated until the final stimulus amplitude and 250 ms nose poke time requirement were reached. Rats then performed FFF versus SSS discrimination using final-amplitude stimuli for 1–4 wk. At this point, the chronic recording drive was implanted, rats rested for 1 week of recovery, and then training was re-initiated until performance regained pre-surgical levels, usually about a week. Finally, FMS and SMF stimuli were added (rewarded right and left, respectively). All behavioral and neural data reported in the study were collected during this final stage.

### FSFS-SFFS Discrimination Task

In this task, each whisker sequence consisted of four pulses. Two pulses were low-amplitude, slow pulses (S) that were 0.7 mm amplitude, 120 mm/sec peak velocity, 12.5 ms rise and fall time, and 25 ms total duration. Two were higher-amplitude, fast pulses (F) that were 1.2 mm amplitude, 216 mm/sec peak velocity, 9 ms rise and fall time, and 18 ms total duration. Trains of F-S-F-S or F-S-S-F pulses were presented (34 ms inter-pulse interval, total train duration 188 ms). In the “same-intensity” stimulus set, both FSFS and SFFS trains had identical pulse amplitude and, therefore, mean speed (mean speed 25.7 mm/sec for FSFS, and 26.4 mm/sec for SFFS). In the “different intensity” stimulus set, FSFS stimulus amplitude (and velocity) was increased to achieve a mean speed of 27.8 mm/sec, and SFFS stimulus amplitude (and velocity) was decreased to achieve a mean speed of 8.7 mm/sec. Training was performed in identical steps as above, using the “different-intensity” stimuli at the second training stage. No recordings were performed.

### Neural Recordings

Recordings were made with an array of four tetrodes carried in a custom 3D-printed chronic microdrive. Tetrodes (12.5 μm nichrome wire, gold plated to 0.2–0.3 MΩ impedance) were spaced 0.35 mm apart in a square configuration and moved together as a single bundle along a radial penetration. The tetrode drive was mounted in a surgical procedure under initial ketamine-xylazine anesthesia (90 mg/kg and 10 mg/kg), maintained by transition to 0.5%–3% isoflurane. A 4-mm craniotomy was opened over S1 (5.5 mm lateral, 2.5 mm caudal to bregma), the dura was removed, and the microdrive was positioned over the durotomy. The tetrodes were lowered into L2 of S1 and the microdrive was mounted with dental cement, sealing the craniotomy. Reference and ground electrodes were mounted in the skull. Postoperative analgesia was provided with Buprenorphine (0.05 mg/kg every 8 h) for 1–2 d post-surgery. Animals recovered 5–10 d prior to behavioral and recording sessions.

Recordings were made during one to two behavioral sessions per day for each rat. Tetrode signals were amplified and filtered (Plexon, 100x gain, 0.3–8 kHz bandpass filter) and digitized at 32 kHz, using methods as in [21]. Neural data was acquired continuously. Tetrodes were advanced a half-turn (140 μm) every one to two recording sessions, at least 30 min before recording started. A new set of units was sampled in every session. If new units appeared spontaneously overnight, the tetrode was not advanced. Recording ended when the tetrode entered the white matter, as judged by absence of spiking activity when advancing the drive. Twelve to 22 d of recording were performed per animal.

Recordings were made in C1–4, D2–4, and E3 whisker columns, as determined by hand mapping under isoflurane anesthesia prior to the recording sessions. An electrolytic lesion was made at the final recording location to determine recording depth. Lesions were recovered in cytochrome oxidase-stained histological sections (100 μm thick) cut in the “across-row” plane, 45° coronal to the midsagittal plane [52,53]. This allowed the whisker row identity (A–E) of the recorded column and laminar identity of recording sites to be confirmed. Laminar boundaries were determined by aligning lesions with layer-specific CO staining boundaries, and were as follows: L2/3: 200–650 μm, L4: 650–975 μm; L5A: 975–1285 μm; L5B: 1285–1575 μm; L6: 1575–2200 μm.

Single units were isolated offline using Wave_clus in Matlab [54]. After an initial automated clustering step, manual evaluation of all clusters was performed and manual changes to the clustering were carried out as needed. Single units were required to meet an interspike interval criterion (<0.5% of intervals less than 1.5 ms) and a signal-to-noise (STN) criterion for spike height (STN>2, with STN defined as the difference from trough to peak in the mean waveform divided by the average standard deviation across all samples in the waveform). Fast-spiking and regular-spiking units were classified by spike width, which was bimodally distributed. Fast spiking units had width <0.375 ms trough-peak delay.

### Neural Data Analysis

Neural data were analyzed for five rats, including one rat for whom the fixed-panel control task showed substantial task performance in the absence of panel movement (filled circles in Fig 2D). This rat’s data were included because panel-evoked responses, stimulus decoding, and choice decoding did not differ from other rats (not shown).

#### Temporal response modulation

We identified units whose firing rate was significantly temporally modulated during the stimulus presentation period (0–180 ms after NP entry) using a permutation test [55]. Measured firing rate was compared in 10-ms bins with randomly time-permuted spikes (10,000 permutations). Units with significant difference from permuted data (*p* < 0.05) were considered temporally modulated and were included in further analysis.

#### Stimulus-evoked responses

PSTHs were calculated with 1 ms time bins, aligned to onset of the first impulse. Unit PSTHs were smoothed (10 ms boxcar) for display only (Fig 4). Stimulus-evoked firing modulation quantifies the peak evoked response in a 40-ms window post-stimulus. It was calculated as the difference between mean baseline firing rate (0–10 ms prior to pulse onset) and maximum or minimum firing rate anywhere in a 40 ms window after stimulus onset (with 10 ms smoothing). Peak response latency was defined as the time of this maximum response. Mean impulse-evoked firing rate was quantified in a 5–35 ms window after impulse onset. Impulse-responsive units were defined as those neurons whose mean impulse-evoked firing rate was significantly greater than baseline firing rate (0–10 ms before impulse onset) by *t* test.

#### Stimulus regression

We performed a multiple regression to determine the optimal stimulus integration window for each unit. The neural responses from 0 to 180 ms relative to stimulus onset were binned into 5 ms windows and used as the dependent variable in this regression. The independent variables (regressors) were the integrated speed of the panel over a series of fixed integration windows, from 5 to 180 ms in 5-ms steps. Each speed bin (e.g., from -20 to 0 ms in the 20 ms integration window regression) was used to predict firing rate in the subsequent 5-ms bin (from 0 to 5 ms in this example). For cells that had significant regressions in at least one stimulus integration window (*p* < 0.05/36 = 0.0014, Bonferroni correction for 36 integration windows tested), the best fit integration window was taken as the stimulus integration window with the highest R^2^ value. Regression was performed in Matlab. Integration window is not independent from latency in this analysis; however, inspection of PSTHs shows that units identified by the regression as having progressively longer best integration windows exhibited progressively slower whisker-evoked responses, not just longer latencies (Figs 7A and 7B, 8A and 8B).

### Neural Decoders

A neural decoder was constructed to predict stimulus identity (FFF, FMS, SMF, SSS) from single-trial spike trains of the recorded units. Each unit was represented by a one-versus-all (OVA) classifier that was trained by logistic regression to report the probability of each stimulus given a single-trial spike train (0–150 ms after stimulus onset, binned using either 10 ms bins or a single fixed time bin), selected randomly from recorded spike trains for that unit. Each classifier comprised four logistic functions, one for each stimulus. Logistic functions were fit using logistic regression and k-fold cross-validation and were specified by coefficients (one for each time bin, plus a bias term) that relate spike rate in each time bin to the probability of stimulus *s* being delivered. Model fitting was performed using a randomly chosen subset of the recorded trials (70%), and decoder performance was assessed on the remaining trials. The output of each unit classifier was normalized so that each unit had the same weight in population decoding. The population stimulus prediction *s*_*p*_ was calculated by summing the probabilities of each stimulus over all units and selecting the stimulus with the maximal summed probability. Model fitting and population decoding were repeated 300 times, and average performance is reported. This framework is equivalent to determining *s*_*p*_ as the stimulus that maximizes the conditional probability of the four stimuli given the neural population response, assuming that all single units are independent and the prior distribution of *s* is uniform. Rate-normalized and time-scrambled spike trains were generated by dividing each spike train by its -Euclidean norm and shuffling spike times within trials, respectively.

A separate behavioral choice decoder was constructed similarly and was used for predicting right or left drink port choice on a given trial. Since this is a binary decision, a single logistic function was fit for each unit. The model was fit using spike train and behavioral choice data from all four stimuli. Decoder performance was assessed separately for FFF, FMS, SMF, or SSS stimulus trials in order to dissociate stimulus identity from the rat’s behavioral choice. The population choice prediction *c*_*p*_ was selected as the choice with maximal summed probability across all units, given single-trial spike trains from trials with the chosen stimulus type. Model fitting and decoding procedures were the same as above. All decoding analysis was performed using Python and the scikit-learn machine learning toolbox [56].

## Supporting Information

S1 Data Data for Figs 1, 2, 3, 6, and 9. (XLSX)

S2 Data Data for S1, S2, and S6 Figs. (XLSX)

S1 Fig Speed profile of each stimulus. Position and speed profiles for FFF, FMS, SMF, and SSS stimuli. Data are in S2 Data.
(TIF)

S2 Fig Additional analysis of FFF-FMS-SMF-SSS behavior. A, Behavior performance was stable over 8–22 d of training. Left, average performance for five rats that had 8–13 d of training. Right, three rats that had 15–22 d of training (2-day bins were used because of low number of animals). Points are mean ± SEM. B, Behavioral effect of fixed panel trials, assessed using a simple alternative to d-prime. FFF versus SSS discrimination was quantified as (fraction of right choices to FFF stimuli–fraction of right choices to SSS stimuli). Discrimination was reduced on fixed panel trials (*p* = 0.012, two-sided paired *t* test). One rat (filled) was not significantly impaired, suggesting that he based discrimination on inadequately masked auditory cues. C, Varied responses to fixed-panel trials across rats. The plot shows fraction of right-side choice for FFF and SSS moving panel stimuli, and for the average of all fixed-panel stimuli. Only the five rats who showed behavioral impairment in the fixed panel session are included. Three rats treated fixed panel stimuli like SSS; one rat chose right or left nearly at random (50% right-side choice); and one rat did not complete trials during fixed-panel blocks. Data are in S2 Data.
(TIF)

S3 Fig Firing rate for single units by layer. A, Firing rate distributions for RS single units (left) and FS single units (right). Both temporally modulated and non-modulated units are included. Open triangles, median. Filled triangle, mean. Arrowhead, mean for multi-unit clusters, shown for comparison. Note different firing rate scales for RS and FS units. B, Firing rate (mean ± SEM) for temporally modulated and non-modulated RS single units. Temporally modulated units generally had higher firing rates, even during the baseline period.
(TIF)

S4 Fig Adaptation for Fast units. A, Panel-evoked responses to each individual impulse within FFF, FMS, SMF, and SSS trains, measured as firing rate 5–35 ms after impulse onset, above pre-sequence baseline. Points are mean ± SEM across all Fast RS units. Adaptation is evident in FFF but not SSS trains. B, Adaptation quantified by paired pulse ratio during FFF and SSS trains. For the same units as in (A). Paired pulse ratio is defined as panel-evoked firing rate during Pulse N/Pulse 1. Error bars, SEM.
(TIF)

S5 Fig Responses to panel impulses by L5 Slow Positive units. A, Mean evoked firing during each impulse (defined as in S4 Fig), for all Slow Positive units in L5a and L5b. Left- and right-choice trials were analyzed separately. Error bars are SEM. Right-choice trials had higher firing rate than left-choice trials, consistent with Fig 8. Firing during pulse 3 did not differ between FFF, FMS, SMF, and SSS stimuli, for either left- or right-choice trials. B, Firing during pulse 3 did not correlate with overall mean sequence speed or with mean speed of the preceding two pulses.
(TIF)

S6 Fig High-speed video analysis of head and whisker movements during the stimulus presentation period. A, Schematic of a rat in the nose poke with head and whisker position measured during each 8.4-ms frame of a single trial, from 0 to 150 ms after stimulus onset. Nose poke entry occurred 50–75 ms prior to stimulus onset. Circles show measured positions of two points on the head (which were pins on a skull-mounted Omnetics connector) and one whisker tip. Nose poke center position is shown at top. We calculated head angle, head position relative to its starting position, and whisker tip position relative to the head. B, Mean trajectories of each variable across 22 left-choice and 21 right-choice trials (n = 4 rats). Bars are SEM. Significant right-choice versus left-choice differences were found for the x position (right-left position) of the head, but for no other variable. Data are in S2 Data.
(TIF)

S7 Fig Contribution of RS and FS units to response classes. A, Prevalence of RS single units, FS single units, and multi-unit clusters within each response class. B, Population PSTHs for Fast units that were RS single units, FS single units, or multi-unit clusters. Each color trace is a different sequence (FFF, FMS, SMF, SSS). Conventions as in Fig 4D. Bars show onset of individual impulses. C, Population PSTH for Slow Positive units in L5 that were RS single units or multi-unit clusters. There were no FS units in this response class. Traces are mean for all four sequences, shown separately for right- and left-choice trials.
(TIF)

S1 Table Firing rates by layer and unit type. These data include both temporally modulated and non-modulated units.
(DOCX)

## Abbreviations

F: fast
FS: fast spike
M: medium
OVA: one-versus-all
PSTH: peri-stimulus time histogram
RS: regular spike
S: slow
S1: primary somatosensory cortex
