# Correction: Short Time-Scale Sensory Coding in S1 during Discrimination of Whisker Vibrotactile Sequences

**DOI:** 10.1371/journal.pbio.1002568

**Published:** 2016-09-28

**Authors:** The PLOS Biology Staff

An error was introduced in Table 1 during the production process. Three of the four headings in section 2 of the table were incorrect. The correct section 2 headings should be listed as follows: Interpulse Interval (ms), Total Sequence Duration (ms), Mean Speed (mm/s), Target Drink Port.

Please see below for the corrected version.

**Table 1:** Kinematics of FFF-FMS-SMF-SSS whisker sequences.

**Impulse**	**Rise/Fall Time (ms)**	**Peak Velocity (mm/s)**	Duration* (ms)	**Peak Amplitude (mm)**
Fast (F)	8	220	16	1.03
Medium (M)	11	170	22	1.15
Slow (S)	14	110	28	1.14
**Sequence**	**Interpulse Interval (ms)**	**Total Sequence Duration (ms)**	**Mean Speed (mm/s)**	**Target Drink Port**
FFF	34	120	65.7	Right
FMS	34	132	54.4	Right
SMF	34	132	54.7	Left
SSS	34	148	44.6	Left

* Duration measured as time from initial deflection to return to baseline position.
