# Supplementary figures and images for: Short Time-Scale Sensory Coding in S1 during Discrimination of Whisker Vibrotactile Sequences
Source: PLoS Biol. 2016 Aug 30;14(8):e1002549. doi: 10.1371/journal.pbio.1002549 (PMC5004814; doi:10.1371/journal.pbio.1002549)

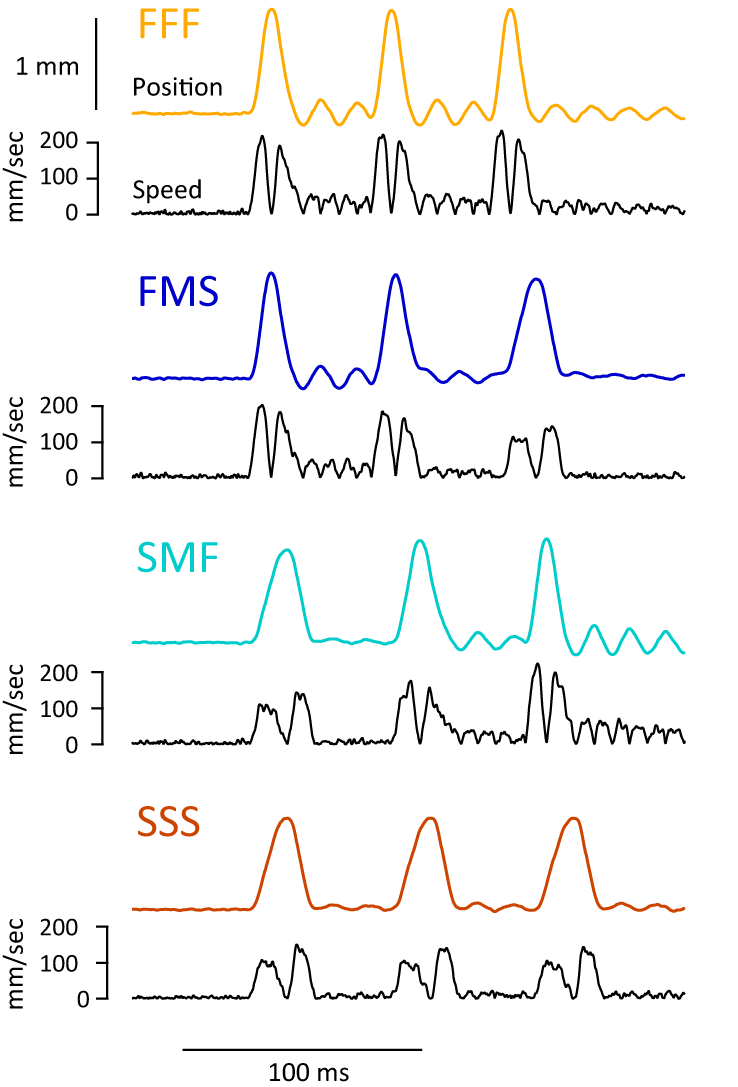

Supplement: S1 Fig — Position and speed profiles for FFF, FMS, SMF, and SSS stimuli. Data are in S2 Data. (TIF) [file pbio.1002549.s003.tif]

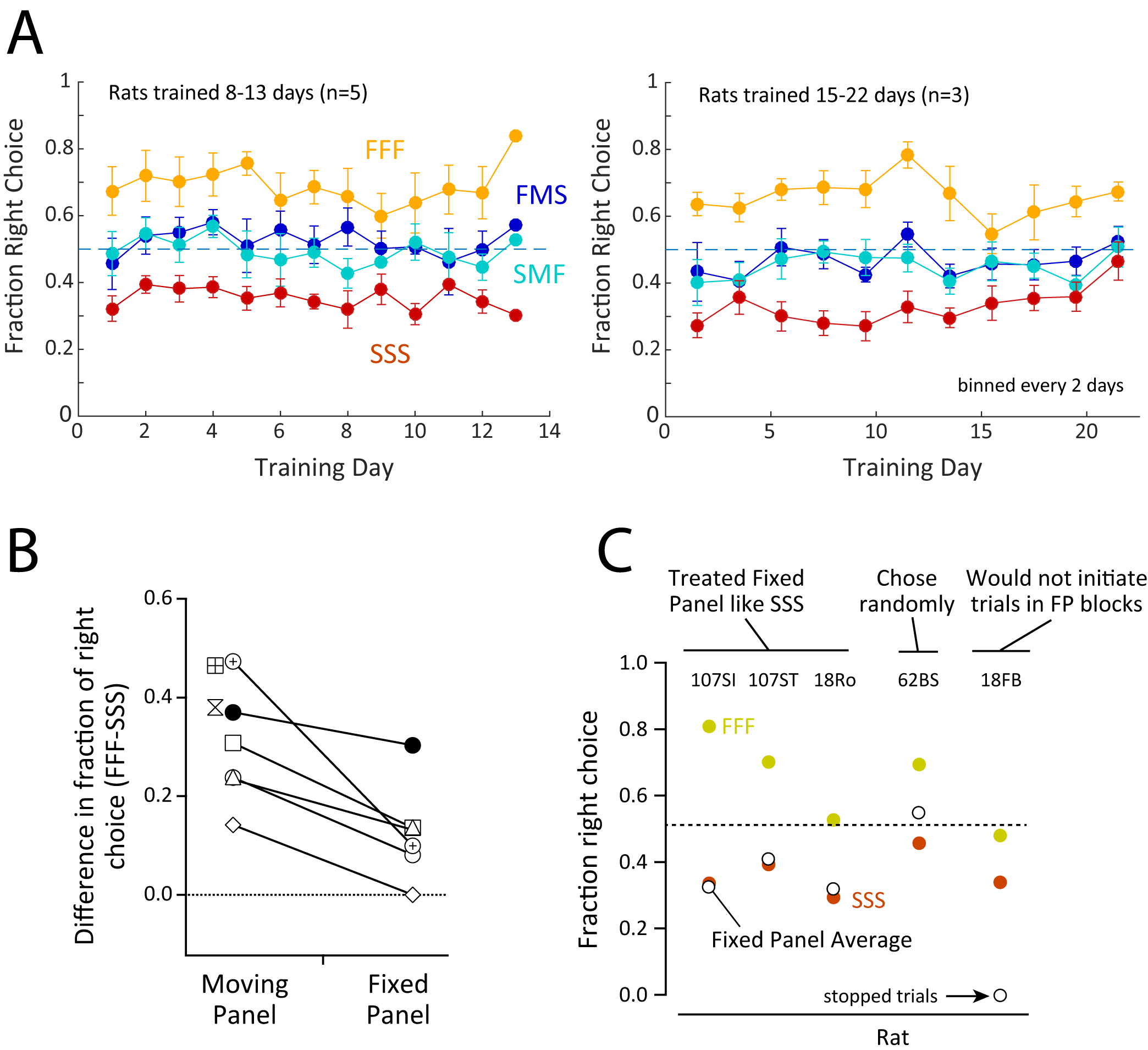

Supplement: S2 Fig — A, Behavior performance was stable over 8–22 d of training. Left, average performance for five rats that had 8–13 d of training. Right, three rats that had 15–22 d of training (2-day bins were used because of low number of animals). Points are mean ± SEM. B, Behavioral effect of fixed panel trials, assessed using a simple alternative to d-prime. FFF versus SSS discrimination was quantified as (fraction of right choices to FFF stimuli–fraction of right choices to SSS stimuli). Discrimination was reduced on fixed panel trials (p = 0.012, two-sided paired t test). One rat (filled) was not significantly impaired, suggesting that he based discrimination on inadequately masked auditory cues. C, Varied responses to fixed-panel trials across rats. The plot shows fraction of right-side choice for FFF and SSS moving panel stimuli, and for the average of all fixed-panel stimuli. Only the five rats who showed behavioral impairment in the fixed panel session are included. Three rats treated fixed panel stimuli like SSS; one rat chose right or left nearly at random (50% right-side choice); and one rat did not complete trials during fixed-panel blocks. Data are in S2 Data. (TIF) [file pbio.1002549.s004.tif]

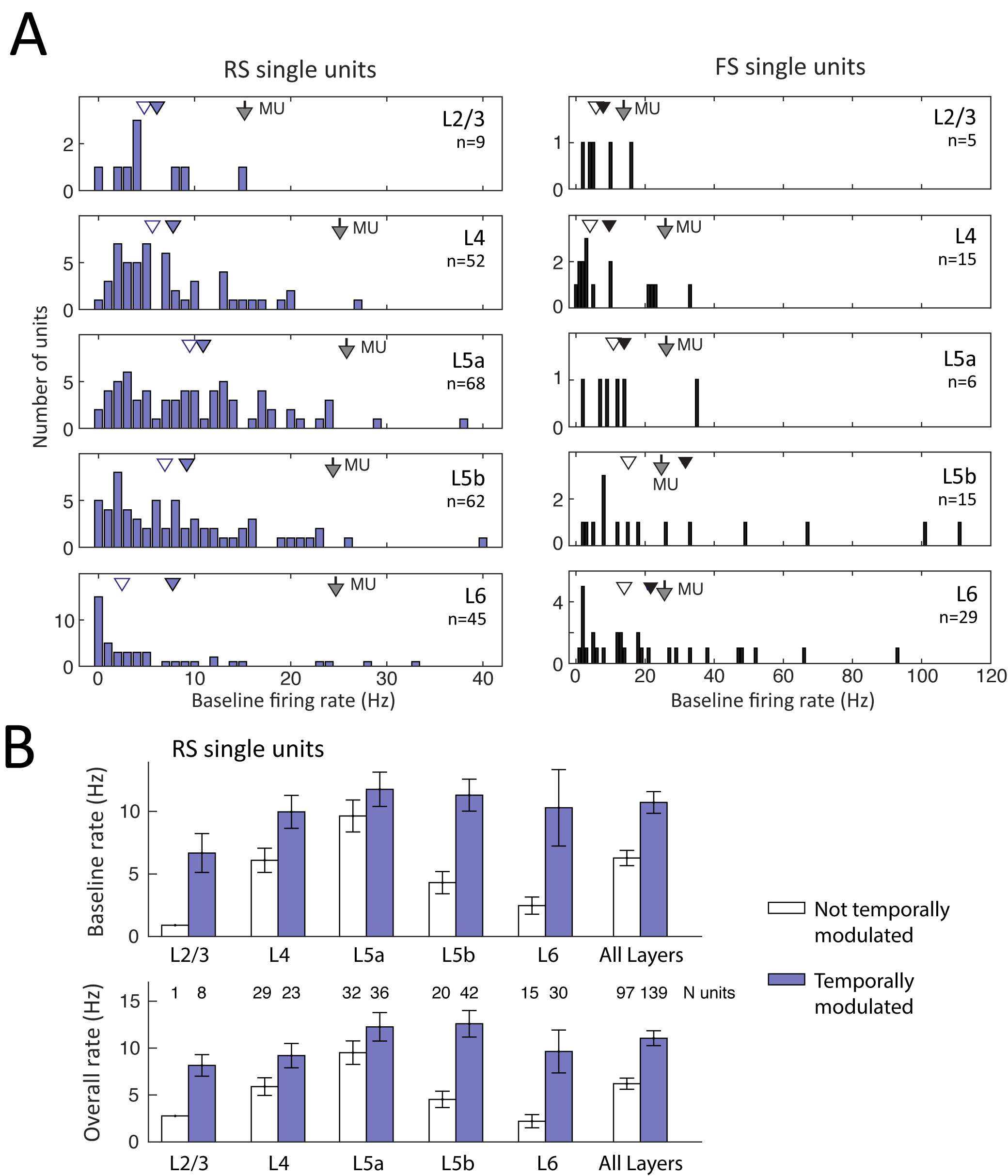

Supplement: S3 Fig — A, Firing rate distributions for RS single units (left) and FS single units (right). Both temporally modulated and non-modulated units are included. Open triangles, median. Filled triangle, mean. Arrowhead, mean for multi-unit clusters, shown for comparison. Note different firing rate scales for RS and FS units. B, Firing rate (mean ± SEM) for temporally modulated and non-modulated RS single units. Temporally modulated units generally had higher firing rates, even during the baseline period. (TIF) [file pbio.1002549.s005.tif]

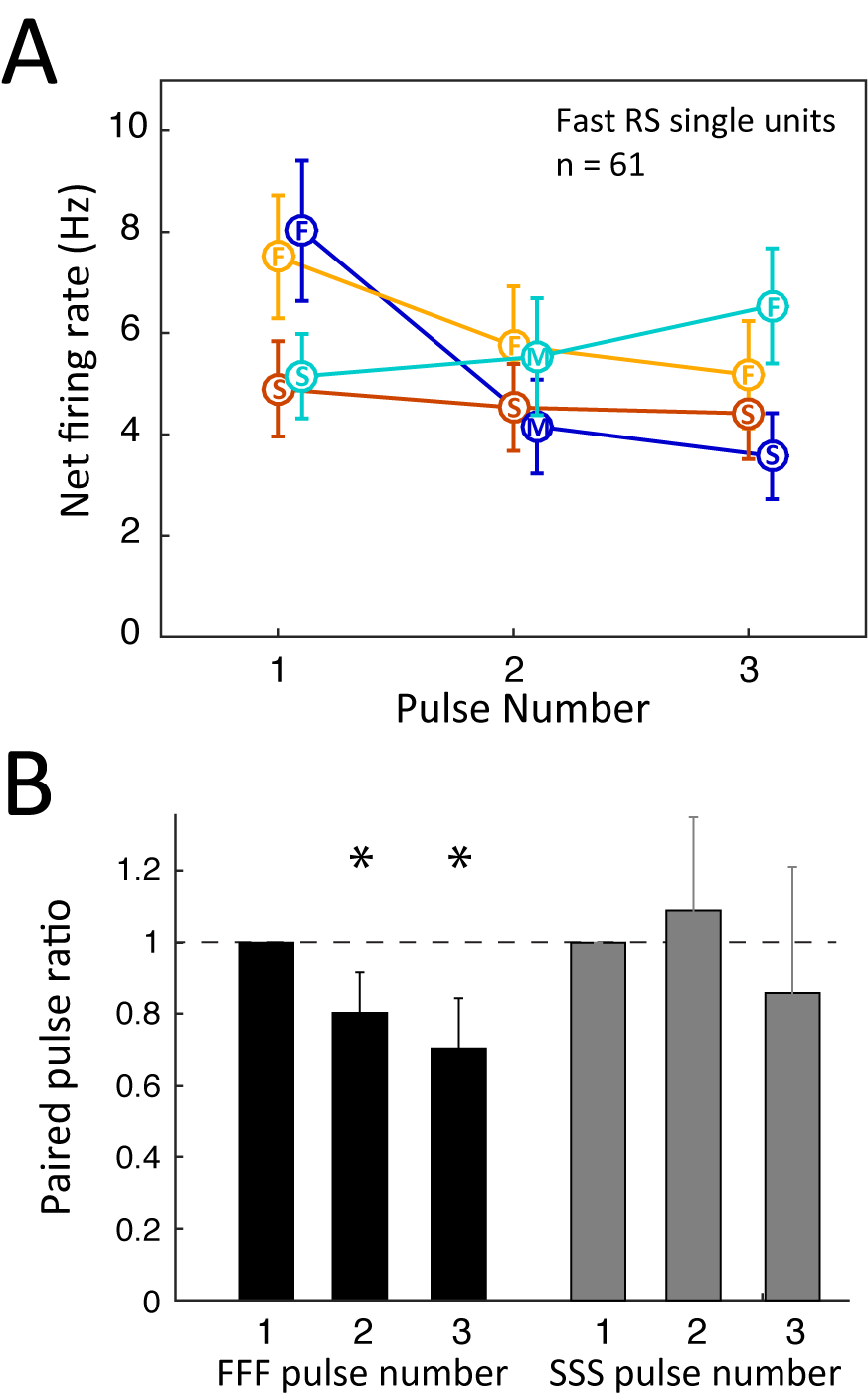

Supplement: S4 Fig — A, Panel-evoked responses to each individual impulse within FFF, FMS, SMF, and SSS trains, measured as firing rate 5–35 ms after impulse onset, above pre-sequence baseline. Points are mean ± SEM across all Fast RS units. Adaptation is evident in FFF but not SSS trains. B, Adaptation quantified by paired pulse ratio during FFF and SSS trains. For the same units as in (A). Paired pulse ratio is defined as panel-evoked firing rate during Pulse N/Pulse 1. Error bars, SEM. (TIF) [file pbio.1002549.s006.tif]

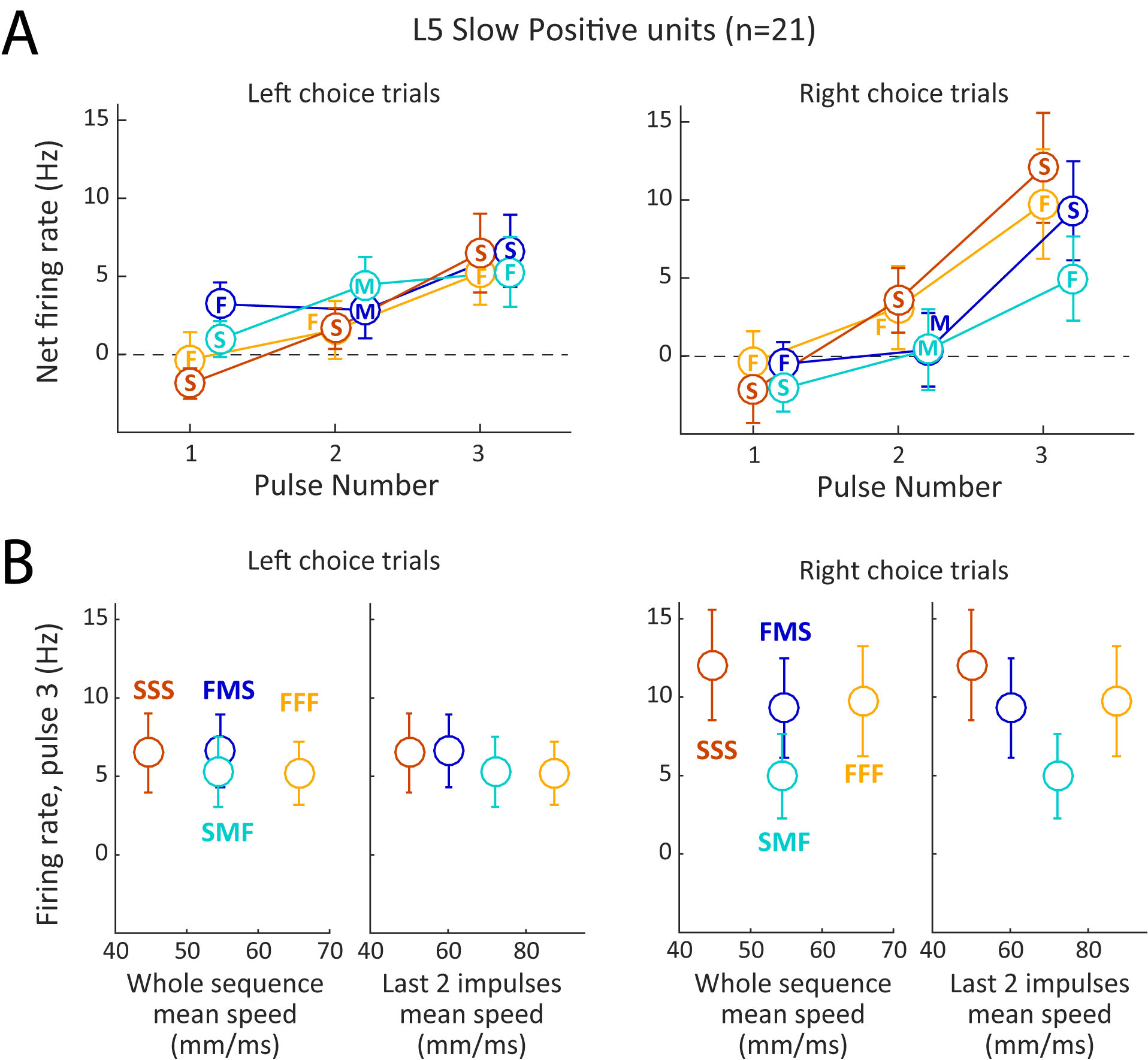

Supplement: S5 Fig — A, Mean evoked firing during each impulse (defined as in S4 Fig), for all Slow Positive units in L5a and L5b. Left- and right-choice trials were analyzed separately. Error bars are SEM. Right-choice trials had higher firing rate than left-choice trials, consistent with Fig 8. Firing during pulse 3 did not differ between FFF, FMS, SMF, and SSS stimuli, for either left- or right-choice trials. B, Firing during pulse 3 did not correlate with overall mean sequence speed or with mean speed of the preceding two pulses. (TIF) [file pbio.1002549.s007.tif]

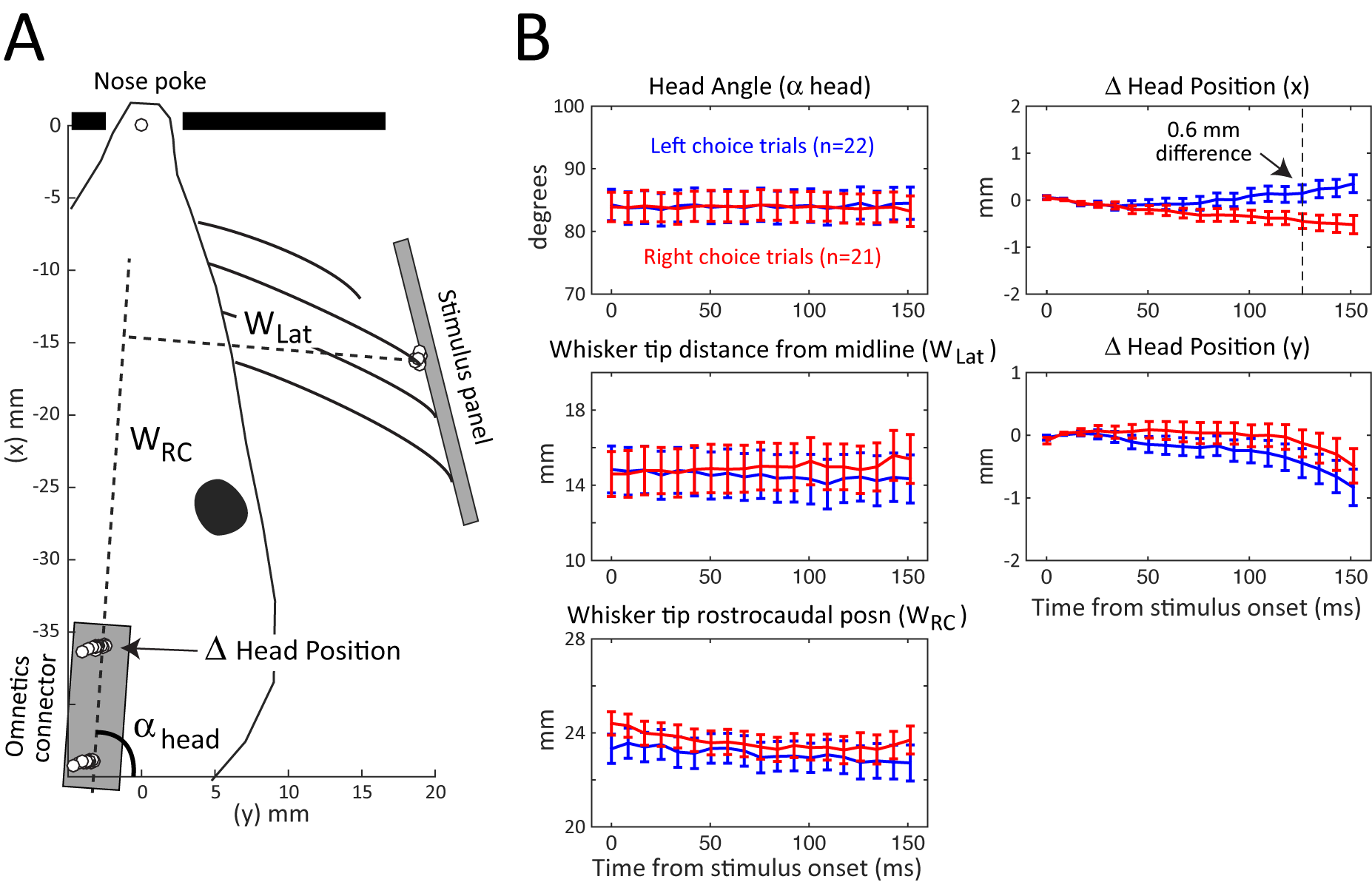

Supplement: S6 Fig — A, Schematic of a rat in the nose poke with head and whisker position measured during each 8.4-ms frame of a single trial, from 0 to 150 ms after stimulus onset. Nose poke entry occurred 50–75 ms prior to stimulus onset. Circles show measured positions of two points on the head (which were pins on a skull-mounted Omnetics connector) and one whisker tip. Nose poke center position is shown at top. We calculated head angle, head position relative to its starting position, and whisker tip position relative to the head. B, Mean trajectories of each variable across 22 left-choice and 21 right-choice trials (n = 4 rats). Bars are SEM. Significant right-choice versus left-choice differences were found for the x position (right-left position) of the head, but for no other variable. Data are in S2 Data. (TIF) [file pbio.1002549.s008.tif]

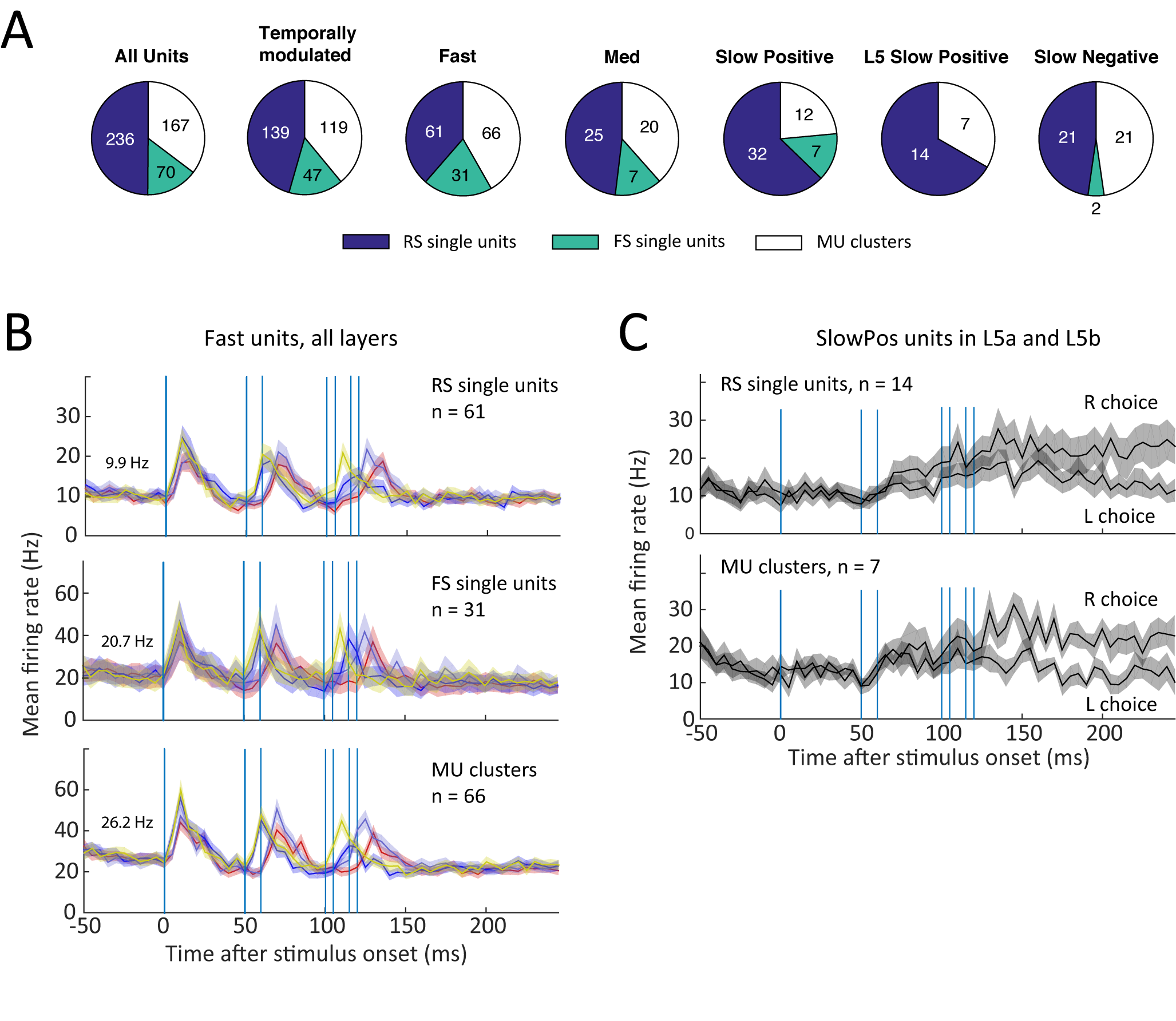

Supplement: S7 Fig — A, Prevalence of RS single units, FS single units, and multi-unit clusters within each response class. B, Population PSTHs for Fast units that were RS single units, FS single units, or multi-unit clusters. Each color trace is a different sequence (FFF, FMS, SMF, SSS). Conventions as in Fig 4D. Bars show onset of individual impulses. C, Population PSTH for Slow Positive units in L5 that were RS single units or multi-unit clusters. There were no FS units in this response class. Traces are mean for all four sequences, shown separately for right- and left-choice trials. (TIF) [file pbio.1002549.s009.tif]
