# Supplementary material for: Short Time-Scale Sensory Coding in S1 during Discrimination of Whisker Vibrotactile Sequences
Source: PLoS Biol. 2016 Aug 30;14(8):e1002549. doi: 10.1371/journal.pbio.1002549 (PMC5004814; doi:10.1371/journal.pbio.1002549)
Supplement: S1 Table — These data include both temporally modulated and non-modulated units. (DOCX) [file pbio.1002549.s010.docx]

|  |  | **Baseline Firing Rate (Hz)** | | | **Average Firing Rate (Hz)** | | |  |
| --- | --- | --- | --- | --- | --- | --- | --- | --- |
| **Layer** | **Type** | **Mean ± SEM** | **Median** |  | **Mean ± SEM** | **Median** |  | **N units** |
| L23 | RS | 6.0 ± 1.5 | 4.9 |  | 7.6 ± 1.2 | 8.1 |  | 9 |
| L23 | FS | 7.9 ± 2.4 | 5.7 |  | 9.0 ± 1.6 | 10.4 |  | 5 |
| L23 | MU | 15.9 ± 3.1 | 14.5 |  | 17.8 ± 3.3 | 17.3 |  | 23 |
|  |  |  |  |  |  |  |  |  |
| L4 | RS | 7.8 ± 0.8 | 5.7 |  | 7.4 ± 0.8 | 5.1 |  | 52 |
| L4 | FS | 9.8 ± 2.7 | 3.9 |  | 10.9 ± 2.8 | 6.9 |  | 15 |
| L4 | MU | 25.3 ± 2.9 | 20.9 |  | 25.8 ± 2.8 | 18.8 |  | 51 |
|  |  |  |  |  |  |  |  |  |
| L5a | RS | 10.8 ± 0.9 | 9.6 |  | 11.0 ± 1.0 | 9.0 |  | 68 |
| L5a | FS | 10.7 ± 4.8 | 10.9 |  | 12.6 ± 5.6 | 7.6 |  | 6 |
| L5a | MU | 26.6 ± 3.1 | 25.8 |  | 25.6 ± 2.9 | 23.9 |  | 30 |
|  |  |  |  |  |  |  |  |  |
| L5b | RS | 9.0 ± 1.0 | 7.0 |  | 10.0 ± 1.1 | 8.4 |  | 62 |
| L5b | FS | 31.6 ± 9.1 | 15.2 |  | 34.0 ± 8.9 | 15.8 |  | 15 |
| L5b | MU | 23.3 ± 2.9 | 20.7 |  | 23.1 ± 2.3 | 20.2 |  | 31 |
|  |  |  |  |  |  |  |  |  |
| L6 | RS | 7.7 ± 2.1 | 2.4 |  | 7.2 ± 1.6 | 3.3 |  | 45 |
| L6 | FS | 21.6 ± 4.1 | 13.8 |  | 22.0 ± 4.3 | 14.8 |  | 29 |
| L6 | MU | 25.2 ± 4.4 | 15.5 |  | 22.9 ± 3.7 | 14.9 |  | 32 |
